# Mitochondria-Microbiota Interaction in Neurodegeneration

**DOI:** 10.3389/fnagi.2021.776936

**Published:** 2021-12-23

**Authors:** Peter Kramer

**Affiliations:** Department of General Psychology, University of Padua, Padua, Italy

**Keywords:** microbiota, mitochondria, Alzheimer’s disease, Parkinson’s disease, gut-blood barrier, butyrate, hydrogen sulfide, methionine

## Abstract

Alzheimer’s and Parkinson’s are the two best-known neurodegenerative diseases. Each is associated with the excessive aggregation in the brain and elsewhere of its own characteristic amyloid proteins. Yet the two afflictions have much in common and often the same amyloids play a role in both. These amyloids need not be toxic and can help regulate bile secretion, synaptic plasticity, and immune defense. Moreover, when they do form toxic aggregates, amyloids typically harm not just patients but their pathogens too. A major port of entry for pathogens is the gut. Keeping the gut’s microbe community (microbiota) healthy and under control requires that our cells’ main energy producers (mitochondria) support the gut-blood barrier and immune system. As we age, these mitochondria eventually succumb to the corrosive byproducts they themselves release, our defenses break down, pathogens or their toxins break through, and the side effects of inflammation and amyloid aggregation become problematic. Although it gets most of the attention, local amyloid aggregation in the brain merely points to a bigger problem: the systemic breakdown of the entire human superorganism, exemplified by an interaction turning bad between mitochondria and microbiota.

## The Roots of Alzheimer’s and Parkinson’s Disease

If we live long enough, we will degenerate; and because it demands a great deal of oxygen and energy, our brain’s decline is particularly conspicuous (Kramer and Bressan, 2018). The most notable neurodegenerative diseases are Alzheimer’s and Parkinson’s, which are so common that in 2015 they affected, respectively, 46 and 6 million people worldwide (Dorsey et al., 2007; GBD 2015 Disease and Injury Incidence and Prevalence Collaborators, 2016; Dorsey and Bloem, 2018; see also Alzheimer’s Association, 2019). On the face of it, the two diseases look quite different. The most notable symptoms of Alzheimer’s are progressive loss of memory and a worsening cognitive ability (Alzheimer’s Association, 2019). The most notable ones of Parkinson’s are tremors, stiffness, and instability when standing or walking (Kalia and Lang, 2015). Often, however, patients suffer from a range of symptoms that do not all neatly fit the diagnostic criteria of one disease or another, and often these patients are concurrently plagued by multiple health issues (Del Tredici and Braak, 2020).

### Both Diseases Feature Excessive Amyloid Aggregation

Underneath, moreover, the two disorders involve broadly similar physiological processes, with a major role in both for amyloids – sticky proteins that can aggregate into nanoscale-tiny fibers (fibrils) and larger clumps. The aggregation is due to the folding of the amyloids in a way that causes similar nearby ones to fold in a similar way – a process akin to the propagation of prion amyloids in mad-cow disease and in its human counterpart, Creutzfeldt-Jakob disease (Goedert, 2015; Stopschinski and Diamond, 2017; but cf. Surmeier et al., 2017; Steiner et al., 2018; Kowalski and Mulak, 2019; Jankovska et al., 2021; Nguyen et al., 2021).

**FIGURE 1:**
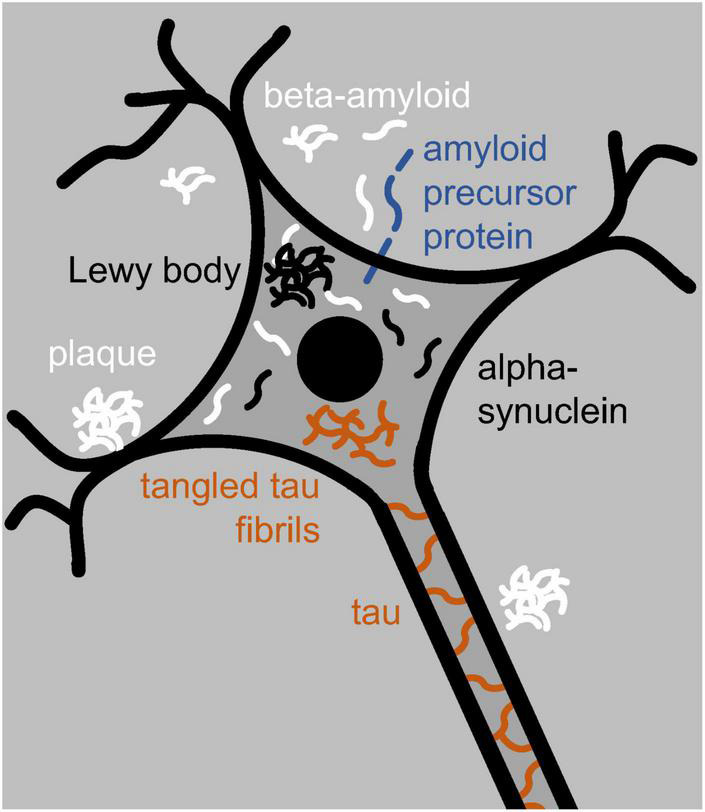
Neuron with a nucleus (black disk), dendrites (black branching), and an axon (running toward the bottom right). The neuron contains tau and tangled-tau fibrils (brown), alpha-synuclein and Lewy bodies (black), and beta-amyloid (white). Outside the neuron are beta-amyloid and amyloid plaques. Locked in the neuron’s membrane is amyloid precursor protein (blue) from which beta-amyloid is cut out.

Soluble amyloid aggregates tend to be toxic; larger insoluble ones, although quite prominent, need not be (Bendor et al., 2013; D’Andrea, 2016; Li et al., 2018; Quntanilla and Tapia-Monsalves, 2020). In Parkinson’s, the soluble amyloid alpha-synuclein can aggregate, inside neurons, into insoluble Lewy bodies (Figure 1; Del Tredici and Braak, 2020). In Alzheimer’s, soluble beta-amyloid can do so into insoluble plaques outside neurons (Figure 1; Makin, 2018). Beta-amyloid is also present inside neurons, and while soluble, it can likely enter neurons from the outside (Bayer and Wirths, 2010; D’Andrea, 2016). Inside neurons, both alpha-synuclein (Guo et al., 2013) and beta-amyloid (Choi et al., 2014) can induce the soluble amyloid tau to aggregate into insoluble tangled fibrils of tau-compounds or truncated pieces of tau (Guo et al., 2017; Quntanilla and Tapia-Monsalves, 2020; Figure 1).

Beta-amyloid, and especially tau, compounds have primarily been associated with Alzheimer’s disease; alpha-synuclein compounds and Lewy bodies instead with Parkinson’s disease (Del Tredici and Braak, 2020; Nguyen et al., 2021). Yet all three amyloids, and additional ones besides, can trigger one another’s aggregation, and mixed neurodegenerative disease is quite common (Nguyen et al., 2021). Alpha-synuclein, for example, contributes to the aggregation of beta-amyloid and tau compounds, tau compounds contribute to the aggregation of alpha-synuclein (Pan et al., 2021), and Lewy bodies have been observed not just in Parkinson’s but in other neurodegenerative disease as well (Bendor et al., 2013; Wong and Krainc, 2017). Moreover, besides alpha-synuclein, Lewy bodies can contain tau and be surrounded by tau fibrils (Pan et al., 2021). And conversely, besides beta-amyloid, plaques typically contain other amyloids (D’Andrea and Nagele, 2010), including alpha-synuclein (Bendor et al., 2013; Jankovska et al., 2021). In addition, the *MAPT* gene, which expresses tau, is implicated in not only Alzheimer’s (Zhou and Wang, 2017) but also Parkinson’s (Pan et al., 2021). It might thus be a mistake to treat the various degenerative diseases of the brain and body as categorically distinct rather than as interrelated (see also Nguyen et al., 2021).

### Both Diseases Feature Poor Mitochondria and a Poor Microbiota

**FIGURE 2:**
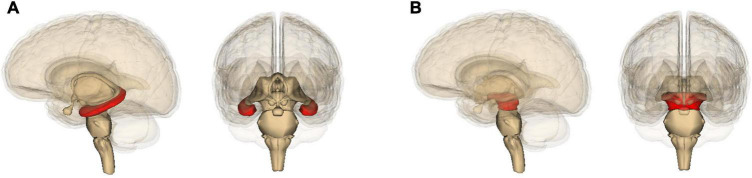
Location in the brain of **(A)** the hippocampus and **(B)** the midbrain (containing the substantia nigra), shown in red. The images are generated by Life Science Databases from the Anatomography website maintained by Life Science Databases (Creative Commons): https://commons.wikimedia.org/w/index.php?curid=7887124; https://commons.wikimedia.org/w/index.php?curid=7837965.

Although both Alzheimer’s and Parkinson’s can progress toward dementia, accompanied by widespread damage (D’Andrea, 2016; Weil et al., 2017), they differ in which part of the brain they damage the most. Alzheimer’s is best known for degeneration of the hippocampus (Figure 2A) and resulting deficits in the formation of new memories (D’Andrea, 2016); Parkinson’s with that of the pars compacta portion of the substantia nigra (Figure 2B) and resulting deficits in one’s ability to move about (Dickson et al., 2009; Poewe et al., 2017). Yet these neurological differences actually highlight yet again how much the two diseases have in common. Most of the energy our body and brain run on is furnished by our mitochondria – entities inside our cells that evolved from bacteria (Lane, 2015). And it so happens that the hippocampus contains unusually low levels of a protein – implicated in Alzheimer’s disease (Acker et al., 2019) – that helps neurons and their mitochondria obtain oxygen (Burmester et al., 2000). This, then, leaves these hippocampal mitochondria particularly vulnerable to dysfunction (Acker et al., 2019). Likewise, the pars compacta hosts exceptionally large neurons, which require exceptionally large amounts of energy, and these neurons’ mitochondria may therefore also be particularly vulnerable to dysfunction (Pacelli et al., 2015; Rietdijk et al., 2017; Shlevkov and Schwarz, 2017; Gonzaìlez-Rodriìguez et al., 2021). This is especially the case because calcium levels in these neurons tend to be higher than elsewhere, and this stimulates alpha-synuclein buildup (Surmeier et al., 2017). In Parkinson’s, much smaller neurons in nearby brain areas tend to be spared (Pacelli et al., 2015; Rietdijk et al., 2017; Shlevkov and Schwarz, 2017).

Although also heavily implicated in virtually every other mental affliction, from chronic psychological stress and fatigue to schizophrenia and autism (Kramer and Bressan, 2018), mitochondrial dysfunction typically both accompanies and precedes Alzheimer’s and Parkinson’s disease (see section “Poor Mitochondria Means a Poor Microbiota”). Such mitochondrial dysfunction can have various different causes, but one of them is an abnormal presence of amyloids (Wong and Krainc, 2017; Bernal-Conde et al., 2020; Huang et al., 2020; Quntanilla and Tapia-Monsalves, 2020; Szabo et al., 2020; Borsche et al., 2021; Feng et al., 2021; Nguyen et al., 2021; Zaretsky and Zaretskaia, 2021). The question arises, however, what causes this abnormal presence. An important part of the answer, it appears, is infection and inflammation (see section “The Main Problem Is Pathogens, Not Amyloids”).

Pathogens can invade us through various points of entry, but a major one is the gut. Primarily in the intestines, we house an estimated 38 trillion microbes (Sender et al., 2016), among which at least 2,172 known species (Hugon et al., 2015), comprising about as many (small) microbial cells as we have (large) human ones (Sender et al., 2016). In addition, these intestines leave space for an even more diverse and numerous collection of viruses (Cryan et al., 2019). Most of these are phages and prey on our gut microbes without affecting us directly (Cryan et al., 2019) but they nonetheless have been reported to affect their host’s gut-blood barrier (Fitzgerald et al., 2019). Even at the best of times, the community of microbes (microbiota) in the gut is not a harmless community of symbionts: we possess a protective gut-blood barrier to keep them out of our bloodstream, and because that is not good enough, we also have a blood-brain barrier to keep them out of our brain. To ease nutrients through, the gut-blood barrier is only one-cell-thick and semipermeable (Groschwitz and Hogan, 2009). In compensation, the intestine (especially the large one, the colon, in which most of the gut microbiota resides) is coated with a layer of sticky mucus, which helps protect the gut-blood barrier from pathogen invasion (von Martels et al., 2017; Kowalski and Mulak, 2019). Most importantly, the barriers of both the small and the large intestine are protected by a heavy presence of the immune system (Kowalski and Mulak, 2019).

This system, however, can only do its job if it is furnished with sufficient energy, a task entrusted mostly to mitochondria. Counterintuitively, some of these mitochondria have a sort of brake on their energy production and release these brakes only when needed (see section “Poor Mitochondria Means a Poor Microbiota”). If the mitochondria fail to deliver the energy the immune system needs, an unhealthy change in the gut microbiota results, known as dysbiosis (see section “Poor Mitochondria Means a Poor Microbiota”). The health of the microbiota thus depends on that of the mitochondria, but because mitochondria need nutrients and are harmed by pathogens, their health also depends on that of the microbiota. For how long the interaction between mitochondria and microbiota remains beneficial, and we can keep neurodegenerative disease at bay, depends in part on the quantity of food we eat (see section “Mitochondria’s Oxygen Consumption Benefits the Microbiota”). It also depends on how rich this food is in, for example, fibers (see section “The Microbiota’s Fatty Acids Benefit Mitochondria”), fatty acids (see section “The Microbiota’s Fatty Acids Benefit Mitochondria”), and protein (see section “The Microbiota’s Hydrogen Sulfide Can Either Benefit or Harm Mitochondria”). With age, both mitochondria and the microbiota deteriorate, and gut pathogens then have multiple options to either indirectly or directly wreak havoc on the brain (see section “Trouble in the Gut Is Trouble in the Brain”). Indeed, Alzheimer’s (Fulop et al., 2018; Moir et al., 2018; Ashraf et al., 2019; Cryan et al., 2019; Li et al., 2021) and Parkinson’s (Cardoso and Empadinhas, 2018; Nair et al., 2018; Cryan et al., 2019; Lubomski et al., 2019; Huang et al., 2021; Munoz-Pinto et al., 2021), along with many other mental afflictions (Vuong et al., 2017; Cryan et al., 2019), tend to be both accompanied and preceded by not only mitochondrial dysfunction but also dysbiosis and intestinal disease (see also Vogt et al., 2017; Villumsen et al., 2019; Shen et al., 2021).

## The Main Problem Is Pathogens, Not Amyloids

### Amyloids Protect Against Pathogens

In low concentrations, amyloids are not a threat but a blessing, and serve useful physiological functions. Beta-amyloid and alpha-synuclein, for example, are involved in fat metabolism, neurotransmitter release, synaptic plasticity, learning and memory (Golovko et al., 2009; Parihar and Brewer, 2010; Cheng et al., 2011; Bendor et al., 2013; D’Andrea, 2016; Fitzgerald et al., 2019; Panza et al., 2019). Tau has an important role in structural support in neurons and in the transport of mitochondria through axons to synapses in need of energy (Guo et al., 2017). Beta-amyloid has been preserved for at least 400 million years, which would be difficult to understand if it had not contributed to survival (Moir et al., 2018). Most Alzheimer’s or Parkinson’s researchers focus their attention on the production and aggregation of amyloids in and around neurons in the brain. Yet amyloids are also produced in other cell types and body parts (D’Andrea, 2016; Filippini et al., 2019), including various organs, the skin, muscles, blood vessels, and the gut (D’Andrea, 2016; Chalazonitis and Rao, 2018).

Both beta-amyloid (Moir et al., 2018) and alpha-synuclein (Barbut et al., 2019) share several characteristics with substances like LL-37 that have well-established antimicrobial properties. In fact, they have anti-pathogenic properties themselves and they, or their soluble aggregates, boost immunity further by triggering inflammation (D’Andrea, 2016; Park et al., 2016; Fulop et al., 2018; Li et al., 2018; beta-amyloid: Moir et al., 2018; Ashraf et al., 2019; alpha-synuclein: Barbut et al., 2019; Fitzgerald et al., 2019; Kowalski and Mulak, 2019; Li et al., 2021). In Alzheimer’s, beta-amyloid levels are typically rather high in the temporal lobes, which host the hippocampus (Soscia et al., 2010). And tellingly, after being injected into post-mortem samples of temporal lobes, *Candida albicans* (a fungus implicated in intestinal disease) grows less in samples taken from Alzheimer’s patients than in samples taken from people of a similar age without the disease (Soscia et al., 2010).

Likewise, after a brain injection of live *Salmonella typhimurium* (a bacterium implicated in food poisoning), mice engineered to overproduce human beta-amyloid displayed a lower pathogen load in the brain (Kumar et al., 2016). They also lost less weight and survived better than their control littermates (Kumar et al., 2016). Mice engineered to underproduce beta-amyloid survived marginally worse (Kumar et al., 2016). Injection with dead *Salmonella* had no such effects.

**FIGURE 3:**
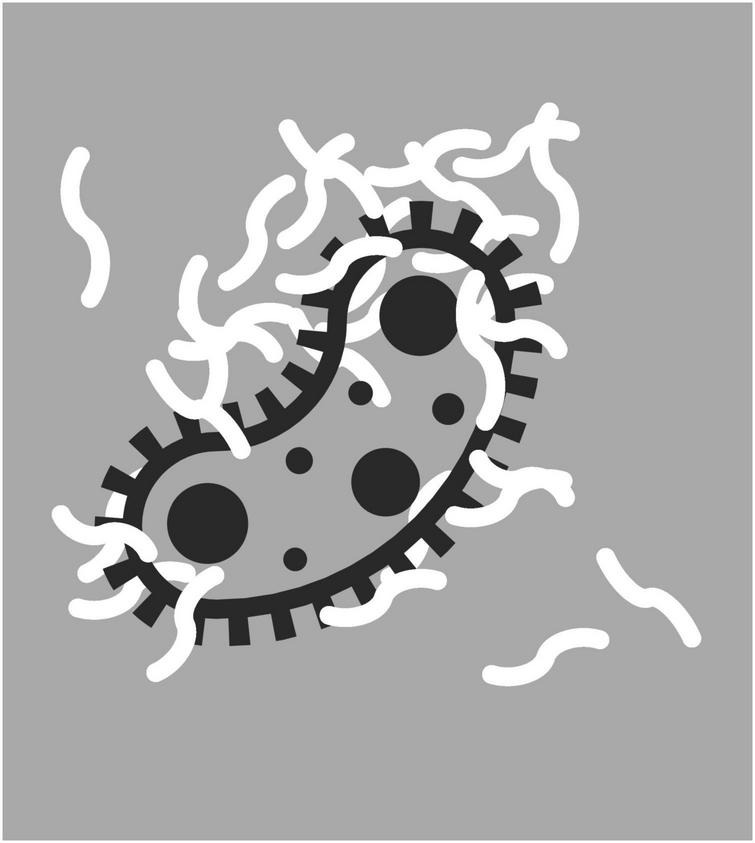
Pathogen entrapped by amyloid fibrils. Icon adapted from the Noun Project: Maxim Kulikov.

Like antimicrobial LL-37, beta-amyloid is sticky, latches on to pathogens like *S. typhimurium*, and entraps them in its fibrils (Figure 3; Kumar et al., 2016). Any antimicrobial property of beta-amyloid is only called for if such pathogens are indeed present. It thus makes sense that even mice that have been engineered to overproduce beta-amyloid have lower levels of beta-amyloid in their brain if they had been raised germ-free than if they had not (Harach et al., 2017; see also Minter et al., 2017). After injection into the bloodstream of live *S. typhimurium*, mice engineered to underproduce alpha-synuclein were also less likely to survive than control littermates (Tomlinson et al., 2017). Infection with a virus, rather than a bacterium, had a similar effect (Beatman et al., 2015; Tomlinson et al., 2017).

Small concentrations of antimicrobial substances like LL-37 provide a net benefit to the host. Large concentrations of its soluble aggregates can instead be toxic and are implicated in several aging-related diseases (Moir et al., 2018). The same is true for the amyloids typically implicated in Alzheimer’s and Parkinson’s (Moir et al., 2018; Barbut et al., 2019). Healthy mitochondria degrade damaged or misfolded proteins, including amyloids like alpha-synuclein and beta-amyloid, but they become overwhelmed when amyloid concentrations become too high (Lautenschäger and Schierle, 2019; Lautenschäger et al., 2020; Sivanesan et al., 2020). Moreover, mitochondria release free radicals – chemicals that are corrosive to their host but also to their own mitochondrial DNA. As a result, over time, more and more of these mitochondria become dysfunctional (Lane, 2015; Kramer and Bressan, 2019) and eventually unable to detoxify the amyloids (Sivanesan et al., 2020).

Inflammation and the formation and aggregation of amyloids and other antimicrobial substances are perhaps best seen as a necessary evil. When moderate and brief, these defenses offer a net benefit to the host and its mitochondria but when excessive and chronic, their side effects may become worse than the infections they were designed to quell (Wong and Krainc, 2017; Bernal-Conde et al., 2020; Huang et al., 2020; Quntanilla and Tapia-Monsalves, 2020; Szabo et al., 2020; Vezzani et al., 2020; Borsche et al., 2021; Feng et al., 2021; Zaretsky and Zaretskaia, 2021). Because amyloid production and aggregation serve a definite purpose, however, suppressing them may be risky. Indeed, drugs that lower beta-amyloid levels raise the risk of infection and have time and again either failed to improve the health of Alzheimer’s patients or made it worse (Green et al., 2009; Imbimbo, 2009; Makin, 2018).

### Bad Genes and Bad Amyloids Offer the Best Protection

By far the greatest risk factor of Alzheimer’s disease, but also implicated in some forms of Parkinson’s disease (Tsuang et al., 2013; Pang et al., 2018; Shi and Holtzman, 2018; Belloy et al., 2019), is the gene *Apolipoprotein E* (*APOE*), which is involved in the transport of cholesterol and other fatty acids or compounds (Urosevic and Martins, 2008; Shi and Holtzman, 2018; Belloy et al., 2019). *APOE* comes in four variants: *APOE1* (extremely rare and not considered further), *APOE2* (rare), *APOE3* (most common), and *APOE4* (common) (Belloy et al., 2019). *APOE4* is evolutionarily the oldest (Belloy et al., 2019). Which *APOE* variant one carries affects tau and alpha-synuclein levels (Huynh et al., 2017; Hohman et al., 2018) and is likely involved in the production, and very clearly in the breakdown, of beta-amyloids (Belloy et al., 2019). Most studies suggest that beta-amyloid buildup is favored least by *APOE2*, a bit more by *APOE3*, and much more by *APOE4* (Belloy et al., 2019). Mirroring this pattern, *APOE2* is the least and *APOE4* the most inflammatory (Moir et al., 2018; Sullivan, 2020).

In Western nations, with a diet rich in inflammatory fats and poor in anti-inflammatory ones and with high blood cholesterol levels (Sullivan, 2020), *APOE4* is a major risk factor for early-onset Alzheimer’s (especially in women: Belloy et al., 2019) and so it has a bad reputation (Sullivan, 2020). Yet in places where, because of a better diet or for other reasons, high cholesterol is less common, *APOE4* is either less of a risk factor for Alzheimer’s (like in East-Asians) or not at all (like in Africa’s Bushmen) (Notkola et al., 1998). The incidence of Alzheimer’s is also higher among Japanese Americans and African Americans than among the Japanese and Africans (here Nigerians) living in their native countries (Sullivan, 2020). And although *APOE4* is a bigger risk factor for Alzheimer’s than is *APOE3*, and Alzheimer’s involves cognitive decline, among infected Tsimane Amazon-Indians, *APOE4* is associated with better cognition than *APOE3* (Trumble et al., 2017; see also van Exel et al., 2017; Zhao et al., 2020). *APOE4*’s more inflammatory effect is a burden in those in good health but apparently can be a boon during infection. More recently, beta-amyloid was found to impair, but *APOE4* itself to slightly improve, short-term memory among some 400 British participants without dementia (Lu et al., 2021).

Beta-amyloid consists of a chain of amino acids that can differ in length, with the most common lengths being either 40 or 42 amino acids (by comparison, alpha-synuclein consists of some 140 amino acids and tau of 352 to 421; Nguyen et al., 2021). Beta-amyloid 42 tends to aggregate into soluble fibrils and insoluble plaques more easily than beta-amyloid 40 and is more toxic. Unsurprisingly therefore, like *APOE4*, beta-amyloid 42 also has a bad a reputation (D’Andrea, 2016; Jankovska et al., 2021). Yet beta-amyloid 42 is not only more toxic to us, it is also more toxic to pathogens (Soscia et al., 2010) and that can be a good thing. Moreover, as mentioned, beta-amyloid has other functions than just helping to fight off infection, and in small amounts both beta-amyloid 40 and beta-amyloid 42 promote neuronal survival (D’Andrea, 2016). What is bad or good about *APOE* variants and amyloids, then, depends on circumstances like diet and pathogen exposure.

The expression of various genes involved in neurodegenerative disease is regulated by epigenetic processes (Balmik and Chinnathambi, 2021) – processes that act *upon* (*epi* in Greek) genes. An important epigenetic process is methylation – the addition of a methyl group to a gene, or nearby protein, so that it can no longer be read. The methyl group is provided by S-adenosyl methionine, a chemical that in part is produced with the help of mitochondria (Wallace and Fan, 2010; Lopes, 2020). Mitochondria are also the prime source of the energy methylation requires. With age, as more and more mitochondria begin to malfunction, this methylation process can go awry. Indeed, diminished methylation can lead to abnormal formation and accumulation of tau compounds (Balmik and Chinnathambi, 2021) and likely of beta-amyloid too (Lin et al., 2009). Thus, even if amyloids help protect us from pathogens, as we age, mitochondrial dysfunction can eventually turn the genes that express these amyloids against us.

## Poor Mitochondria Means a Poor Microbiota

### Keeping the Microbiota Healthy Requires Energy

Before cognitive or motor symptoms emerge, metabolism invariably has already gone awry in both Alzheimer’s (Sun et al., 2020b) and Parkinson’s (Phillipson, 2017), as well as in risk factors of these two diseases: diabetes (Arnold et al., 2018; Cheong et al., 2020), obesity (Floud et al., 2020; Kim and Jun, 2020; Qu et al., 2020), impaired amyloid clearance (Phillipson, 2017; Sun et al., 2020b). Metabolism and amyloid clearance rely to a large extent on mitochondria, which during neurodegeneration are deteriorating (Phillipson, 2017; Sun et al., 2020b; Nguyen et al., 2021). One way to simulate the decline in mitochondrial health in lab animals is to expose mitochondria to a poison. When they are poisoned, mitochondria produce less energy and more free radicals, just like they do during aging. If its cells receive less energy and more free radicals, the gut-blood barrier becomes leaky and lets pathogens through into the bloodstream (Jackson and Theiss, 2020). Lowering free radical levels with antioxidants shores up the barrier, instead, and diminishes its leakiness (Jackson and Theiss, 2020), which helps prevent dysbiosis (Thevaranjan et al., 2017).

**FIGURE 4:**
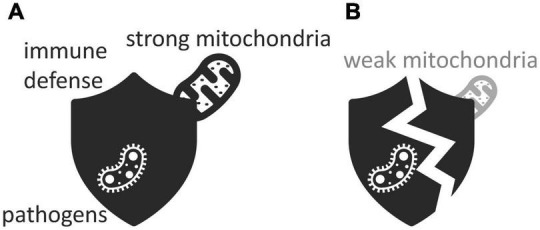
Immune defenses protect against pathogens but only if they are empowered by **(A)** strong mitochondria rather than **(B)** weak ones. Icons adapted from the Noun Project: Maxim Kulikov (bacterium), dDara (mitochondrion), and David Khai (shield).

Mitochondria are not only important for the maintenance of protective barriers but also for the proper functioning of the immune system (Cardoso and Empadinhas, 2018; Munoz-Pinto et al., 2021). Each mitochondrion features an outer membrane that separates it from the rest of the cell, and an inner membrane that contains its machinery for energy production (Kramer and Bressan, 2019). Curiously, the inner membrane of the mitochondria that reside in a particular type of white blood cells produces much less energy than it is capable of Konjar et al. (2018). Upon recognizing viruses, bacteria, or harmful debris, immune cells release proinflammatory signaling molecules that, in defense of the host, trigger inflammation (Jackson and Theiss, 2020). When this happens, the properties of the inner membrane of the white-blood cells’ mitochondria abruptly change: the brakes come off and energy production shoots up (Konjar et al., 2018). This sudden boost of energy allows the white blood cells to proliferate fast and take immediate action against their foes. If mitochondria cannot produce the energy that white blood cells need to fight off infection, the gut-blood barrier suffers, and gut pathogens get a chance to thrive and invade their host’s bloodstream (Figure 4; Konjar et al., 2018). Mitochondrial dysfunction reduces the energy available to the immune system and impairs the immune system’s ability to keep the gut microbiota under control. This, then, leads to dysbiosis as well (Yardeni et al., 2019; Jackson and Theiss, 2020).

### Energy Production Deteriorates With Age

Activation of the immune system comes with a surge of free radicals that is harmful not only to invading pathogens, but also to the tissues of the host itself and the mitochondria in the cells of these tissues (López-Armada et al., 2013; Angajala et al., 2018). Aside from the fact that they consume resources, inflammation and free-radical production should thus not be brought to bear more than strictly needed (Konjar et al., 2018). This, then, might be why mitochondria behave like gate keepers; they hold their guard dogs (immune cells) on a tight leash when all is fine and set them loose only when bad characters (pathogens) come charging in Kramer and Bressan (2019).

**FIGURE 5:**
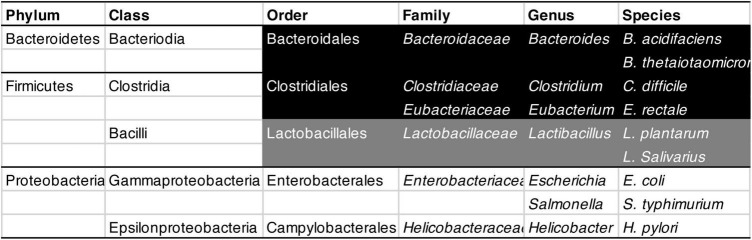
Classification of bacteria mentioned in this review, from Phylum (the widest category) to Species (the narrowest). From Order to Species, strictly anaerobic bacteria are shown in white on black, largely anaerobic ones in white on gray, and aerobic ones in black on white.

Although free radicals can harm mitochondria, the mitochondria release many of them themselves during energy production (Lane, 2015; Kramer and Bressan, 2019). Much of the DNA that mitochondria carried as independent bacteria was eventually incorporated into the nucleus of their host cells (Lane and Martin, 2010; Lane, 2015). Yet the mitochondria did hold on to 37 genes, of which 13 express proteins needed for the mitochondrial energy-production machinery (Johnston and Williams, 2016). To a large extent, mitochondrial genes determine how much energy mitochondria can produce (Johnston and Williams, 2016) and how many free radicals they release in the process (Moreno-Loshuertos et al., 2006, 2013). Lab animals have been bred or engineered that carry versions of mitochondrial DNA that increase free-radical production (Yardeni et al., 2019). The higher this production, the leakier the gut, offering opportunities to gut pathogens (Jackson and Theiss, 2020). In fact, mice made to produce more free radicals harbor a less diverse gut microbiota, with more of it consisting of Bacteroidales (a subset of Bacteroidetes; Figure 5) rather than Clostridia (a subset of Firmicutes; Figure 5; Yardeni et al., 2019). Consistently, after 10 weeks on a drug that lowers rather than raises free radical levels, normal mice ended up with 31% fewer Bacteroidales, and more than twice as many Clostridia, as before (Yardeni et al., 2019).

Bacteroidales need not necessarily be harmful. *Bacteroides acidifaciens*, for example, helps prevent obesity and improve insulin sensitivity (shown in mice: Yang et al., 2017). Conversely, Clostridia need not necessarily be beneficial; once ingested, *Clostridium difficile* can cause food poisoning and kills thousands each year in the US alone^1^ (Czepiel et al., 2019). Most studies do not investigate individual species or strains of bacteria but entire families or other broad categories. Substantial methodological differences between studies complicate matters further, with some studies controlling for medication whereas others do not (Romano et al., 2021). Unsurprisingly, they regularly come to different conclusions about which ones are beneficial or harmful (Cryan et al., 2019). Still, aging – the largest risk factor for both Alzheimer’s and Parkinson’s (Hou et al., 2019) – is generally accompanied by a less diverse microbiota (Cardoso and Empadinhas, 2018; Haran and McCormick, 2021; Romano et al., 2021), and in mice also by a proliferation of Bacteroidales at the expense of Clostridiales (Figure 5; Yardeni et al., 2019).

Alas, improving the gut microbiota requires more than just enriching or replacing it. Mice deficient in tumor necrosis factor (a trigger of inflammation) are protected not only from age-related inflammation and a leaky gut but also from dysbiosis (Thevaranjan et al., 2017). Reducing the levels of this signaling molecule with a drug reproduces some of these positive effects (Thevaranjan et al., 2017). This shows once again that the gut microbiota is affected by the immune system and its signaling molecules. Because the levels of tumor necrosis factor rise with age (Thevaranjan et al., 2017), dysbiosis should be expected to worsen with age, and indeed it does (Thevaranjan et al., 2017). Moreover, inflammation is typically accompanied by a rise in free radical levels, and in mice whose mitochondrial DNA has been engineered to engender excessive free-radical production, revealing effects were obtained with cross-fostering. In this procedure, the offspring of mothers engineered to produce relatively many free radicals are given the microbiota of a foster mother engineered to produce relatively few. Within 2 months, the adopted microbiota changed into one consistent with the biological mother’s and not the foster mother’s (see also Hirose et al., 2017 and for related human evidence: Ma et al., 2014; Yardeni et al., 2019). Keeping a healthy microbiota thus requires keeping healthy mitochondria too, and this means being lucky enough to carry good mitochondrial genes. As we get older, mitochondria accrue more and more mutations, release more and more inflammation provoking debris (Munoz-Pinto et al., 2021), countermeasures to mitigate the harm begin to fail (Lane, 2015), and keeping the microbiota healthy becomes less and less feasible. Of course, if someone’s dysbiosis is due not to genes but to circumstances, such as a poor diet or a treatable infection, hope remains for a more lasting cure.

## Mitochondria’s Oxygen Consumption Benefits the Microbiota

### Metabolism Regulates Gut Oxygen Levels

To function well, mitochondria need to be fed. They can only metabolize food that has been digested. After having been chewed to bits, meals are first broken down with the help of acid in the stomach, and bile and enzymes in the small intestine; next, the process is taken over by the microbiota, mainly in the large intestine. The gut microbiota expands through cloning and with each of our meals; it diminishes with defecation. Among the bacteria frequently found in the gut, the majority by far use little or no oxygen (here collectively referred to as “anaerobes”), a minority can or must use larger quantities of this gas (here collectively referred to as “aerobes”) (von Martels et al., 2017). Perhaps because our well-oxygenated tissues are not ideal places for them, anaerobes are less inclined than aerobes to invade, or interfere with, these tissues (Shelton et al., 2020). Although anaerobes are by no means all harmless symbionts (the Bacteroidales and Clostridia mentioned before are all anaerobes), aerobes are more often associated with disease (Shoaie et al., 2021). A healthy gut is thus one in which anaerobes tend to do well and aerobes do not (Rigottier-Gois, 2013; Byndloss et al., 2017; Litvak et al., 2017).

Gut oxygen levels depend on the kind of metabolism the host engages in Byndloss et al. (2017) and Wang et al. (2019). In each of our cells except red blood cells, the conversion of digested food into energy (catabolism) typically involves mitochondria (Wang et al., 2019). In this process, food is effectively treated as fuel and burned down with the help of lots of oxygen (Wang et al., 2019). Instead, the conversion of food into either building blocks for growth, or fat or glycogen for energy storage (anabolism), occurs mostly without engaging mitochondria, and requires much less oxygen or none at all (Byndloss et al., 2017; Wang et al., 2019). So, cells extract far more oxygen from their surroundings when they catabolize food than when they anabolize it. The consequences are particularly interesting if one looks at the cells that compose the gut wall: when they burn food for energy (rather than fermenting it for building blocks or energy storage), the level of oxygen near the gut wall drops (von Martels et al., 2017), and the microbiota as a whole becomes more anaerobic and healthier (Byndloss et al., 2017; Litvak et al., 2017).

### Fasting Improves Metabolism

**FIGURE 6:**
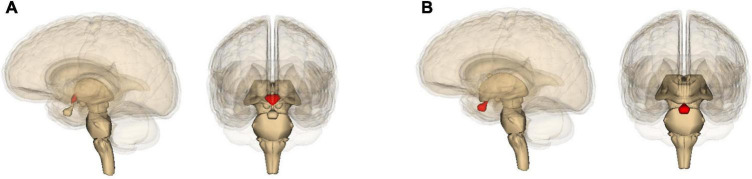
Location in the brain of **(A)** the hypothalamus and **(B)** the pituitary gland, shown in red. Images are generated by Life Science Databases from the Anatomography website maintained by Life Science Databases (Creative Commons): https://commons.wikimedia.org/w/index.php?curid=7848243; https://commons.wikimedia.org/w/index.php?curid=7833196.

Whether food is catabolized or anabolized is regulated in part by hormones sent out by the hypothalamus and an adjacent organ directly under its control: the pituitary gland (Figure 6). In part, it is also regulated inside cells by various chemicals that act like sensors of nutrients, of pathogen invasion, and of other intracellular circumstances (Wang et al., 2019). If the hypothalamus and individual cells ascertain that food is abundant and circumstances suitable, they push for growth and reproduction, which rely heavily on anabolism. If the opposite is the case, they encourage maintenance operations, which rely heavily on catabolism (Wang et al., 2019). For the survival of genes, growth and reproduction are obviously paramount; for the survival of their host, however, maintenance is more important than either growth or reproduction. Likely to allow for growth and reproduction (and for the storage of energy for leaner times), we have evolved to enjoy eating abundantly. Eating no more than necessary, however, has repeatedly been found to improve the health of both mitochondria (Mattson and Arumugam, 2018; Mattson et al., 2018) and the gut microbiota (Rinninella et al., 2020). In addition, fasting lowers free radical levels and inflammation (Mattson and Arumugam, 2018; Mattson et al., 2018), improves leaky gut problems and gut disorders (Rinninella et al., 2020), diminishes overweight and diabetes (Anton et al., 2018), stimulates the release of brain-derived neurotrophic factor (which stimulates the growth of nerves) (Mattson et al., 2018), and helps stave off neurodegenerative diseases like Alzheimer’s and Parkinson’s (Mattson and Arumugam, 2018).

Still, when facing pathogens, the immune system needs not only catabolism to energize immune cells but also anabolism to allow these cells to grow and multiply (Wang et al., 2019; Jackson and Theiss, 2020). Dieting should thus not be so extreme that there are no reserves left to fight infections or for essential growth of, for example, new neuronal connections. Indeed, in elderly individuals and others who suffer disproportionately from infections, inflammation, or weak mitochondria, dieting could be too stressful and hasten death rather than extending life (Forster et al., 2003; Mulvey et al., 2014; Lazic et al., 2020). For everyone else, however, adopting a diet that promotes mitochondrial catabolism and reduces extra-mitochondrial anabolism helps improve the health of the gut microbiota and stave off disease.

## The Microbiota’s Fatty Acids Benefit Mitochondria

### Fatty Acids Nourish Mitochondria and Harm Various Pathogens

Among the beneficial bacteria that in the large intestine thrive on little or no oxygen are many that ferment soluble and insoluble fibers or starches that in the small intestine resist digestion (Sajilata et al., 2006; Canfora et al., 2015; Cryan et al., 2019; Silva et al., 2020; Gill et al., 2021). These fibers and starches can be found in abundance in, for example, fruits and vegetables and cooled-off boiled potatoes. During the fermentation of these fibers, fatty acids are produced by bacteria such as *Bacteroides* (Figure 5), and these fatty acids can then be consumed by the host or by other bacteria that, in turn, produce different fatty acids. *Bacteroides thetaiotaomicron*, for example, ferments dietary fibers and releases the fatty acid acetate; the firmicute *Eubacterium rectale* consumes this acetate and releases the fatty acid butyrate (Shoaie et al., 2013).

Butyrate is a prime fuel for the mitochondria of colon cells, which typically burn it down with lots of oxygen (Donohoe et al., 2011; Byndloss et al., 2017). The low oxygen levels in the gut that result from butyrate catabolism is, as mentioned, particularly harmful to gut pathogens. At the same time, mitochondria that are well-nourished with butyrate should be able to offer better support to the cells of the gut wall and gut-blood barrier (Jackson and Theiss, 2020), which in turn can then better prevent gut pathogens from invading the bloodstream. Whether mitochondria mediate the effect or not, increased butyrate production and exposure has indeed been associated with a better functioning gut-blood barrier (Lewis et al., 2010; Knudsen et al., 2018; but cf. Tabat et al., 2020).

Butyrate affects pathogens in a more direct way as well. The fatty acid consists of a very short chain of carbon atoms, and like other fatty acids of this kind, dissolves both the protective outer layer of viruses and the type of cell-wall that pathogenic microbes tend to have more often than do benign ones (Alcock et al., 2012). Likely because they reduce the need for the immune system to act, very-short-chain fatty acids, and butyrate in particular, have an anti-inflammatory effect (Fernández et al., 2016; Couto et al., 2020). Inflammation relies not only on catabolism but also on anabolism (Wang et al., 2019). So, the toning down of inflammation should, help reduce gut oxygen levels further and create a virtuous cycle.

One of the pathogens directly harmed by butyrate is *Helicobacter pylori* (Yonezawa et al., 2012), which is typically found in the mouth and digestive tract – especially the stomach – of about half the world’s population (Doulberis et al., 2018). *H. pylori* releases a toxin that induces mitochondria of white blood cells to kill their host cells so that they can no longer harm the pathogen (see also Foo et al., 2010; Akazawa et al., 2015). If *H. pylori* is abundant enough, it can cause the side effects of ulcers and stomach cancer (Foo et al., 2010; Akazawa et al., 2015). The bacterium is typically found far from the brain and it is unclear whether it can trigger amyloid deposition (Wang et al., 2015) or not (Albaret et al., 2020). Its eradication fails to improve already existing symptoms of neurodegeneration (Huang et al., 2018; Tan et al., 2020). Still, *H. pylori* does appear to inflame neurons (Albaret et al., 2020) and has been found to raise the risk of Parkinson’s disease (Çamcı and Oğuz, 2016; Shen X. et al., 2017; Huang et al., 2018, 2021). There are indications that, especially in combination with other pathogens (Beydoun et al., 2020), *H. pylori* is also a risk factor for Alzheimer’s and associated conditions (see also Shen L. et al., 2017; Doulberis et al., 2018; but cf. Fani et al., 2018). Injecting *H. pylori* in between the membranes lining the abdominal cavity has been found to lead to learning and memory deficits in rats (Wang et al., 2015).

Short-chain fatty acids not only benefit mitochondria and harm gut pathogens like *H. pylori*, but also have another remarkable effect: they strongly inhibit the aggregation of amyloids, or at least that of beta-amyloid (shown *in vitro*; Ho et al., 2018). The levels of butyrate in feces correlate with those in the brain (shown in mice: Zhang et al., 2017), and tellingly, the feces of patients with Parkinson’s contain abnormally few butyrate-producing bacteria (Unger et al., 2016; Cirstea et al., 2020). Moreover, concentrations of butyrate in feces have been associated not only with Parkinson’s in humans (Unger et al., 2016), but also with aging in humans in general (Duncan and Flint, 2013) and with an Alzheimer-like condition in mice (Zhang et al., 2017). A nutrient that is particularly good for our mitochondria (butyrate) thus happens to be particularly harmful to gut pathogens (like *H. pylori*) and helps prevent or delay neurodegenerative disease.

### Probiotics and Prebiotics Stimulate Fatty Acid Production

The production of fatty acids through bacterial fermentation of fibers in the colon can be improved by consuming probiotics (foods rich in beneficial bacteria such as fermented products like yoghurt or kimchi) and prebiotics (foods rich in soluble or insoluble fibers probiotic bacteria ferment) (LeBlanc et al., 2017). Fatty acids are an important nutrient for the mitochondria of colon cells, but also for those elsewhere. In addition, they can be converted into cholesterol and other fatty substances with a wide range of uses (LeBlanc et al., 2017). One can induce a Parkinson’s-like condition in rats by injecting a poison in their right medial forebrain bundle (Nurrahma et al., 2021). Feeding such rats the probiotic *Lactobacillus salivarius* improves their mitochondrial function, raises antioxidant levels, and prevents deterioration in movement, loss of muscle mass, and loss of dopamine neurons (Nurrahma et al., 2021). A prebiotic has a comparable, but more limited, effect (Nurrahma et al., 2021). Consistently, two somewhat similar studies that induced an Alzheimer’s-like, rather than a Parkinson’s-like, condition showed a protective effect of two probiotic strains of Peng et al., 2014; Divyashri et al., 2015; *Lactobacillus plantarum* Mallikarjuna et al., 2016; see also Cryan et al., 2019 for a review, and in addition Abraham et al., 2019).

A meta-analysis across several studies involving humans failed to find any benefit of probiotics on cognition (Marx et al., 2020). Yet this analysis did not consider the health status of participants and a probiotic might not benefit people who already have a healthy gut microbiota, as the authors themselves point out, or who have already incurred irreparable damage. Probiotics do ameliorate conditions like obesity and diabetes (Hampe and Roth, 2017), which typically involve disturbed metabolism and mitochondrial dysfunction and are risk factors of both Parkinson’s and Alzheimer’s (Arnold et al., 2018; Biosa et al., 2018; Vieira et al., 2018; Chornenkyy et al., 2019; Luca et al., 2019; Rigotto and Basso, 2019; Cheong et al., 2020; Carranza-Naval et al., 2021; Nguyen et al., 2021). Diabetes is, in fact, associated with a reduction in butyrate-producing bacteria (Maniar et al., 2017). And Metformin, a prime drug against diabetes, happens to benefit butyrate-producing bacteria (Hampe and Roth, 2017; Maniar et al., 2017), seems to improve Alzheimer’s disease, and might even delay aging more generally (Maniar et al., 2017). So, healthy mitochondria promote an oxygen-avoiding, butyrate-producing gut microbiota (see section “Mitochondria’s Oxygen Consumption Benefits the Microbiota”), and conversely, such a microbiota also promotes the health of mitochondria.

Given that supplementing diets with pro- or pre-biotics typically increases gut microbial fatty acid production, and that fats are fatty-acid compounds, it is interesting to note that an unsupplemented diet poor in carbohydrates, no more than adequate in protein, but high in fats, has repeatedly been found to produce similar benefits. The diet improves mitochondrial function after brain trauma (Greco et al., 2016) and can prevent or ameliorate cognitive deficits and Alzheimer’s and Parkinson’s disease (but cf. Bostock et al., 2017; Włodarek, 2019; Cunnane et al., 2020; Lilamand et al., 2020; Rawat et al., 2021). The diet is intended to mimic the positive health effects of those that restrict the consumption of food more broadly. Still, for elderly people who are degenerating and have already begun to lose weight, such dieting may no longer be advisable (Włodarek, 2019) (see also section “Mitochondria’s Oxygen Consumption Benefits the Microbiota”).

## The Microbiota’s Hydrogen Sulfide Can Either Benefit or Harm Mitochondria

### Hydrogen Sulfide Can Be a Boon

The highlight of a satisfying meal is typically some source of protein: meat, fish, cheese. Its component amino acids stimulate growth and reproduction at the expense of maintenance operations (Kim and Guan, 2019; Kitada et al., 2021). Essential amino acids like methionine, which are critical to normal growth, are obtained from food with the help of enzymes, the microbiota in the small intestine, and – to a small extent – also the microbiota in the large intestine (Ma and Ma, 2019). Methionine can be recycled from dysfunctional mitochondria or other cellular debris as well (Kitada et al., 2021). A chemical reaction of methionine with adenosine triphosphate (ATP), which is biology’s most important portable energy store, produces S-adenosyl-methionine (SAM), which is biology’s most important molecule for the silencing or expression of genes (Loenen, 2006; Janke et al., 2015; Kramer and Bressan, 2021). In fact, it is not methionine itself but SAM that promotes growth and reproduction and diminishes maintenance and recycling (Kitada et al., 2021). Mitochondria are the most important source of ATP and the production of SAM depends on mitochondria too, and in part on mitochondrial DNA. This means that normal growth depends on the health and genetic makeup of mitochondria (Kramer and Bressan, 2021).

Provided its intake remains sufficient, the less methionine is consumed, the more its metabolization is directed toward the production of glutathione and the gas hydrogen sulfide. Glutathione is an antioxidant and in small quantities so is hydrogen sulfide, but hydrogen sulfide is particularly important as a kind of neurotransmitter (Paul and Snyder, 2018; Kitada et al., 2021; Paul, 2021; Sokolov et al., 2021). The less methionine or SAM and the more hydrogen sulfide is available to a cell, the more of the cell’s dysfunctional mitochondria and other debris are recycled, the better its remaining mitochondria and cellular components perform, and the fewer free radicals its mitochondria release (Paul and Snyder, 2018; Wu et al., 2020; Kitada et al., 2021; Sokolov et al., 2021). This, in turn, allows fatty-acid producing gut bacteria to thrive at the expense of gut pathogens (Wu et al., 2020) and promotes a normal permeability of the *gut-blood* barrier (Ramalingam et al., 2010). In the process, homocysteine derived from methionine is also metabolized (Paul and Snyder, 2018; Kitada et al., 2021), and the resulting low levels of homocysteine have been found to promote the normal permeability of the *blood-brain* barrier (Kamath et al., 2006). Restricting the consumption of protein, and in particular of methionine, improves not only the health of mitochondria, the gut microbiota, and protective barriers, but also the health and lifespan of the host (Soultoukis and Partridge, 2016; Kitada et al., 2021; Sokolov et al., 2021).

Small amounts of hydrogen sulfide are anti-inflammatory (Blachier et al., 2007) and as a result promote the health of both our mitochondria and microbiota. In addition, small amounts of this gas inhibit the formation of abnormal amyloid compounds of alpha-synuclein (Hou et al., 2017), tau (Giovinazzo et al., 2021), and beta-amyloid (Rosario-Alomar et al., 2015) and helps keep at bay neurodegenerative disease (Hou et al., 2017; Paul and Snyder, 2018; Munoz-Pinto et al., 2021; Paul, 2021). Conversely, mutations in genes involved in methionine metabolism, dysregulated methionine metabolism, and abnormal hydrogen-sulfide and glutathione levels have all been implicated in neurodegenerative disease and in aging more generally (Paul, 2021; Sokolov et al., 2021).

### Hydrogen Sulfide Can Be a Burden

Since primordial times, hydrogen sulfide has sustained life (Fu et al., 2012; Lane, 2015; Paul and Snyder, 2018), and in small amounts it still has a net beneficial effect on us today (Blachier et al., 2007; Paul and Snyder, 2018; Kitada et al., 2021; Sokolov et al., 2021). Many people, however, are exposed to larger quantities of this smelly gas, quantities that are more toxic than beneficial (Blachier et al., 2007; Sokolov et al., 2021). Some gut microbes in the colon metabolize either sulfate or – more importantly – sulfur-containing amino acids, which are both obtained from partially digested protein (Blachier et al., 2007). Among the amino acid metabolizers are familiar pathogens like particular strains of *Escherichia coli* and *Salmonella* (Jackson and Theiss, 2020). These microbes are more flexible than many others. They thrive on oxygen but can do without it too. At least one particular strain of pathogenic *E. coli* switches its metabolism during gut inflammation toward the fermentation of amino acids when these are available, whereas more beneficial strains do not. This flexibility gives the pathogen, and others like it, an edge over its less flexible competition (Kitamoto et al., 2020).

During the metabolism by gut bacteria of sulfate or sulfur-containing amino acids, hydrogen sulfide is released, and mitochondria – especially those of colon cells (Goubern et al., 2007) – can use this gas as a fuel (Blachier et al., 2007; Fu et al., 2012). Yet if too much protein has been consumed, which benefits gut pathogens, the concentration of hydrogen sulfide can become harmful to mitochondria (Blachier et al., 2007; Mottawea et al., 2016). As these mitochondria begin to malfunction, butyrate catabolism and energy production decrease; free-radical production, inflammation, gut-blood permeability increase; and intestinal disorders worsen (Blachier et al., 2007; Singh and Lin, 2015; Mottawea et al., 2016; Bajpai et al., 2018; Pal et al., 2018; Aguilar-López et al., 2020; Jackson and Theiss, 2020; Sokolov et al., 2021). Moreover, whereas low levels of hydrogen sulfide have a positive effect on the vascular system (Paul and Snyder, 2018) – and directly or indirectly diminish neuroinflammation, cognitive deficits, and the risk of Alzheimer’s and Parkinson’s – high levels exert the opposite effect (Paul and Snyder, 2018; Paul, 2021).

As an aside, note that like hydrogen sulfide, nitric oxide is also a gas that functions like a neurotransmitter. It is produced by both mitochondria (Litvinova et al., 2015) and the gut microbiota (Tse, 2017), its effects interact with those of hydrogen sulfide (Blachier et al., 2007), and it also has net beneficial consequences in small amounts and harmful ones in large amounts. And in large amounts, it too has been implicated in dysbiosis, mitochondrial dysfunction, and Alzheimer’s and Parkinson’s disease (Litvinova et al., 2015; Tse, 2017).

Let us return to protein, methionine, and hydrogen sulfide. Note that the health benefits that can be obtained through restricting food intake, including delayed neurodegeneration, can also be obtained by simply restricting protein, or even just methionine, intake (see also Wei et al., 2017; Thelen and Brown-Borg, 2020; Wu et al., 2020; Kitada et al., 2021; McCarty and Lerner, 2021). Like protein, vegetables like broccoli and cauliflower contain sulfur and during their digestion hydrogen sulfide is released too (Citi et al., 2014). This release, however, is slow (Citi et al., 2014) and as a result has a positive effect on our health (Whiteman et al., 2010). Broccoli and cauliflower, and members of the Brassicaceae family more generally, also contain sulforaphane. This sulfurous substance is toxic not only to a number of the vegetables’ own microbial enemies (Schillheim et al., 2018) but also to many of our gut pathogens, including *H. pylori* (Kellingray et al., 2017; Le et al., 2020). So, whereas healthy mitochondria promote an oxygen-avoiding, butyrate-producing gut microbiota, and conversely such a microbiota also promotes the health of mitochondria, protein- or methionine-fermenting gut microbiota harms them instead. And this harmful effect has downstream ones on both Alzheimer’s and Parkinson’s.

## Trouble in the Gut Is Trouble in the Brain

### Gut Pathogens’ Interaction With the Brain Is Multipronged

Besides low levels of oxygen and hydrogen sulfide, and high levels of fatty acids, a healthy gut requires the proper regulation of immunity, hormones, gut-blood permeability, bowel movements, and stomach-acid and bile secretion. The orchestration of the full system is demanding enough that the gut has dedicated to this task its own bespoke nervous system. Hinting at its importance, this “enteric” nervous system evolved before the central one did (Snoek et al., 2010; Furness, 2012; Cryan et al., 2019; Niesler et al., 2021) and comprises between 400 and 600 million neurons, covering the entire 32 m^2^ inside surface of the intestines (Helander and Fändriks, 2014).

Dysfunctional gut receptors of the enteric nervous system, as well as genetic aberrations of the enteric nervous system, the central nervous system, and the connections between them, have all been associated with gut disorders (Snoek et al., 2010; Cryan et al., 2019; Niesler et al., 2021). Alzheimer’s (Cui et al., 2018; Waziry et al., 2020) and Parkinson’s (Liu et al., 2020) raise the risk of stroke. Remarkably, this kind of trauma is associated not just with the death of neurons in the brain but also with subsequent degeneration of neurons in the gut (Cheng et al., 2016; Stanley et al., 2016; Blanke et al., 2021) and with dysbiosis (Cryan et al., 2019, 2020). Moreover, zebrafish genetically engineered to grow to maturity without an enteric nervous system become more susceptible to dysbiosis and gut inflammation (Rolig et al., 2017). A transplant of a healthy enteric nervous system corrects this situation, as does a simple probiotic (Rolig et al., 2017). That a probiotic can be effective suggests that the enteric nervous system is less important when the gut microbiota is healthy than when it is not.

**FIGURE 7:**
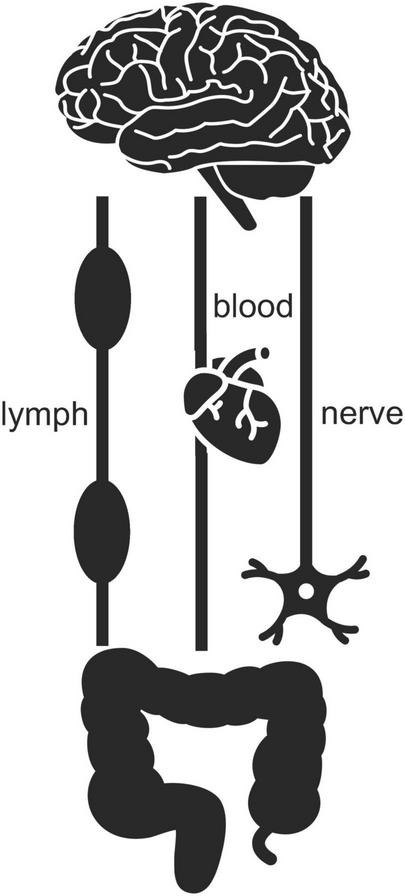
Two-way signaling between gut and brain can proceed via the lymph, blood, and nervous systems. Icons adapted from the Noun Project: Clockwise (brain), Tezar Tantular (heart), public domain (nerve), Bom Symbols (intestine).

The enteric nervous system is part of the autonomous nervous system along with the sympathetic nervous system, which is involved in urgent actions and psychological stress, and the parasympathetic nervous system, which is involved in maintaining homeostasis and calming down stress. The sympathetic and parasympathetic nervous systems connect the enteric nervous system to the central one and enable two-way signaling between them (Figure 7). A pair of parasympathetic nerves, together referred to as the vagus nerve, is particularly important (Bonaz et al., 2017). Gut microbes can affect the firing of this nerve through their production and release of nutrients like fatty acids and vitamins, neurotransmitters like serotonin and dopamine, and various toxins (LeBlanc et al., 2017; Cryan et al., 2019; Fülling et al., 2019). Changes in the composition of the gut microbiota can also, via the vagus nerve, be relayed to the brain by the host’s own enteric neurotransmitters, hormones, and immune signaling molecules (Bonaz et al., 2017; Cryan et al., 2019). Gut viruses and gut microbes that manage to slip or break through protective barriers, or the toxins or other chemicals of gut microbes that do so, can also physically travel up to the brain via either the bloodstream, the lymph system, or the sympathetic and parasympathetic nervous systems – including the vagus nerve (Zhan et al., 2018; Cryan et al., 2019; Fülling et al., 2019; Kowalski and Mulak, 2019; Huang et al., 2021; Figure 7). So, any battle between mitochondria and microbiota in the gut can easily have consequences as far away as in the brain.

### Gut-Bacterial Amyloid Formation Triggers Human Amyloid Formation

Beside their hosts, gut bacteria can produce amyloids too (Friedland and Chapman, 2017; Tursi and Tükel, 2018; Van Gerven et al., 2018; Vidakovic et al., 2018). Curli, for example, is an amyloid produced by for example *E. coli* that binds these bacteria together in a biofilm that helps protect them from viruses and the host’s immune system (Friedland and Chapman, 2017; Fulop et al., 2018; Tursi and Tükel, 2018; Van Gerven et al., 2018; D’Argenio and Sarnataro, 2019; Kowalski and Mulak, 2019; Miraglia and Colla, 2019; Huang et al., 2021; Miller et al., 2021). The bacterial amyloids resemble those of the host and can trigger the folding and clumping of the host’s amyloids. One study compared aged rats of a kind that is particularly susceptible to alpha-synuclein buildup and that were force-fed either Curli-producing *E. coli* or nearly identical control *E. coli* that were incapable of producing Curli. As predicted, those that had received the Curli-producing *E. coli* via the mouth deposited more alpha-synuclein in the brain, and showed more signs of inflammation there, than those who had received the control *E. coli* (see also Lundmark et al., 2005; Chen et al., 2016). A polyphenol found in especially green tea blocks the formation of Curli and biofilm by *E. coli* and reduces alpha-synuclein aggregation and deposition in the striatum and midbrain of *E. coli*-infected mice that had been genetically engineered to overproduce human alpha-synuclein (Sampson et al., 2020). Consumption of the polyphenol reduced both gastrointestinal and motoric deficits.

After injecting bacterial amyloids into the wall of the stomach of mice, aggregates of fluorescent mouse beta-amyloid were found within hours in the enteric nervous system, within a month elsewhere in the gut as well, and within a year in the vagus nerve and brain, including the hippocampus (Sun et al., 2020a). The spread of beta-amyloid from the stomach to the brain subsequently compromised both spatial short-term, and spatial long-term, memory (see also Choi et al., 2018; Sun et al., 2020a).

Broadly similar results were obtained when aggregates of alpha-synuclein rather than beta-amyloid were orally administered or injected into various locations in and around the gut, with the alpha-synuclein affecting also specifically the substantia nigra (see also Braak et al., 2003; Holmqvist et al., 2014; Rietdijk et al., 2017; Barbut et al., 2019; Kim et al., 2019; Lohmann et al., 2019; Van Den Berge et al., 2019). One study investigated mice that had been engineered to overexpress human alpha-synuclein and that, in addition, had been raised germ free (Sampson et al., 2016; see also Sampson, 2020). Of these mice, those given a (microbe rich) fecal transplant from Parkinson’s patients, rather than healthy people, ended up with more dysbiosis, intestinal problems, and Parkinsonian motor deficits. Mice that did not overexpress human alpha-synuclein were unaffected by a such a fecal transplant. And mice that were not raised germ free benefited from antibiotic treatment. In line with these findings, human Parkinson’s patients with bacterial overgrowth in the small intestine show reduced Parkinsonian symptoms after antibiotic treatment (Fasano et al., 2013). Skepticism remains (Munoz-Pinto et al., 2021). Indeed, live imaging of mitochondrial function and calcium levels in neurons contained in small-intestine biopsies did not reveal any difference between Parkinson’s patients and their unaffected partners (Desmet et al., 2017). No differences with regard to alpha-synuclein were found either. With only 15 couples as participants, the study might have lacked sufficient power. Several other studies, however, also failed to find a difference between patients and controls in alpha-synuclein accumulation in the gut (Munoz-Pinto et al., 2021).

Even in young, healthy people, potentially harmful aggregates of alpha-synuclein are often found in the appendix (Killinger et al., 2018) – a protrusion of the colon that first and foremost serves as a safe house for beneficial bacteria (Bollinger et al., 2007). When illness harms the gut microbiota, the bacteria from the appendix can repopulate the gut and, if these bacteria are beneficial, they can help restore the health of the gut microbiota (Bollinger et al., 2007). Still, because the appendix can also harbor pathogens, its removal can sometimes have a positive effect too. An epidemiological study involving more than 1.6 million participants – and thus definitely not underpowered – showed, in fact, that removing the appendix diminishes the risk of Parkinson’s decades later, especially in people from rural areas who may have been more exposed than others to dysbiosis-causing pesticides (Killinger et al., 2018). The procedure also delayed Parkinson’s by up to several years among those who eventually did develop the disease (Killinger et al., 2018).

Severing the vagus nerve, rather than removing the appendix, can also hinder the propagation of alpha-synuclein from the gut to the brain in mice (Kim et al., 2019) and humans (Svensson et al., 2015) and improve a Parkinson’s-like affliction and Parkinson’s itself in, respectively, animals and humans (Borghammer, 2018; Liddle, 2018; Lionnet et al., 2018; Fitzgerald et al., 2019; Kim et al., 2019; Munoz-Pinto et al., 2021). This does not always happen, however, and overall the evidence so far is still mixed (Borghammer, 2018; Lionnet et al., 2018). Removal of the vagus nerve, of course, still leaves the bloodstream and lymph system open to amyloids, pathogens, or their toxins, and this could have confounded results. Indeed, in baboons, injected alpha-synuclein has been found to be transported from the gut to the brain and vice versa via the bloodstream (Arotcarena et al., 2020).

The bloodstream is affected early on in both Alzheimer’s (D’Andrea, 2016) and Parkinson’s (Berganzo et al., 2013; Pierzchlińska et al., 2021), so much so in fact that it has been argued that Alzheimer’s may be just as much a vascular disease as a neurological one (D’Andrea, 2016; Fulop et al., 2018). At least in Parkinson’s, however, the vascular problems are in part related to a disturbance of circadian rhythms, with high blood pressure being a problem particularly at night rather than during the day (Berganzo et al., 2013). Circadian rhythms are, in fact, disturbed in both Parkinson’s (Liu et al., 2021) and Alzheimer’s (Uddin et al., 2020; Sharma et al., 2021), are intertwined with cyclic changes in mitochondrial metabolism (Kramer and Bressan, 2019; Aguilar-López et al., 2020), and affect – and are affected by – the composition of the microbiota (Aguilar-López et al., 2020; Bishehsari et al., 2020; Sen et al., 2021). In sum, gut microbes need not necessarily leave the gut to trigger amyloid deposition in the brain. Whether this happens depends in part on mitochondrial metabolism and circadian rhythms. And to close the circle, these circadian rhythms are under the control of a central biological clock that is located in the brain (Kramer and Bressan, 2019; Aguilar-López et al., 2020).

## Conclusion: Neurodegeneration Is Systemic Degeneration

At first sight, Alzheimer’s and Parkinson’s seem quite distinct diseases; they strike different areas of the brain in different ways and produce different behavioral deficits. It has been shown, for example, that experimentally inducing a specific dysfunction within the mitochondria (involving inner-membrane Complex I) of a specific kind of neuron (the type that uses dopamine as a neurotransmitter) in a specific part of the brain (the substantia nigra, and likely just the pars compacta part of it) is sufficient to induce progressively worsening symptoms of Parkinson’s disease that are not shared with Alzheimer’s disease (Gonzaìlez-Rodriìguez et al., 2021). Yet in natural settings, outside the lab, many patients suffer from a mix of the two diseases, with similar mitochondrial dysfunction affecting multiple cell types; with amyloid aggregates associated with either disease often found in the other as well; with similar accompanying infections, inflammation, and dysbiosis; and with similar effects of diet, lifestyle, trauma, and exposure to toxicants. Naturally developing Alzheimer’s and Parkinson’s may more accurately be described as different manifestations of one and the same systemic problem that involves not just certain areas of the brain, not just the brain as whole, not just the body as a whole, but the entire human superorganism, including even the non-human entities that reside inside us (see also Kramer and Bressan, 2015). Early players in neurodegeneration are deteriorating aerobic ex-bacteria (mitochondria), located throughout our body and brain, and thriving aerobic bacteria (unhealthy microbiota), located mostly as far away from the brain as the gut. Despite the diversity in neurodegenerative diseases, there is a common theme among them: a synergy between mitochondria and microbiota early in life is sooner or later disturbed and, late in life, turned into its very opposite.

## Author Contributions

The author confirms being the sole contributor of this work and has approved it for publication.

## Conflict of Interest

The author declares that the research was conducted in the absence of any commercial or financial relationships that could be construed as a potential conflict of interest.

## Publisher’s Note

All claims expressed in this article are solely those of the authors and do not necessarily represent those of their affiliated organizations, or those of the publisher, the editors and the reviewers. Any product that may be evaluated in this article, or claim that may be made by its manufacturer, is not guaranteed or endorsed by the publisher.

## Footnotes

1
https://www.cdc.gov/hai/organisms/cdiff/cdiff_infect.html

## References

[B1] Abraham D. Feher J. Scuderi G. L. Szabo D. Dobolyi A. Cservenak M. (2019). Exercise and probiotics attenuate the development of Alzheimer’s disease in transgenic mice: Role of microbiome. *Exp. Gerontol.* 115 122–131. 10.1016/j.exger.2018.12.005 30529024

[B2] Acker Z. P. V. Luyckx E. Dewilde S. (2019). Neuroglobin expression in the brain: A story of tissue homeostasis preservation. *Mol. Neurobiol.* 56 2101–2122. 10.1007/s12035-018-1212-8 29992530

[B3] Aguilar-López B. A. Moreno-Altamirano M. M. B. Dockrell H. M. Duchen M. R. Sánchez-García F. J. (2020). Mitochondria: an integrative hub coordinating circadian rhythms, metabolism, the microbiome, and Immunity. *Front. Cell Dev. Biol.* 8:51. 10.3389/fcell.2020.00051 32117978 PMC7025554

[B4] Akazawa Y. Matsuda K. Isomoto H. Matsushima K. Kido Y. Urabe S. (2015). BH3-only protein Bim is associated with the degree of *Helicobacter* pylori-induced gastritis and is localized to the mitochondria of inflammatory cells in the gastric mucosa. *Int. J. Med. Microbiol.* 305 553–562. 10.1016/j.ijmm.2015.07.002 26197709 PMC4556732

[B5] Albaret G. Sifré E. Floch P. Laye S. Aubert A. Dubus P. (2020). Alzheimer’s disease and *Helicobacter* pylori infection: Inflammation from stomach to brain. *J. Alzheimers Dis.* 73 801–809. 10.3233/JAD-190496 31868664

[B6] Alcock J. Franklin M. L. Kuzawa C. W. (2012). Nutrient signaling: Evolutionary origins of the immune-modulating effects of dietary fat. *Q Rev. Biol.* 87 187–223. 10.1086/666828 22970557

[B7] Alzheimer’s Association. (2019). 2019 Alzheimer’s disease facts and figures. *Alzheimers Dem.* 15 321–387. 10.1016/j.jalz.2019.01.010

[B8] Angajala A. Lim S. Phillips J. B. Kim J.-H. Yates C. You Z. (2018). Diverse roles of mitochondria in immune responses: novel insights into immuno-metabolism. *Front. Immunol.* 9:1605. 10.3389/fimmu.2018.01605 30050539 PMC6052888

[B9] Anton S. D. Moehl K. Donahoo W. T. Marosi K. Lee S. A. Mainous I. I. I. A. G. (2018). Flipping the metabolic switch: understanding and applying the health benefits of fasting. *Obesity* 26 254–268. 10.1002/oby.22065 29086496 PMC5783752

[B10] Arnold S. E. Arvanitakis Z. Macauley-Rambach S. L. Koenig A. M. Wang H.-Y. Ahima R. S. (2018). Brain insulin resistance in type 2 diabetes and Alzheimer disease: concepts and conundrums. *Nat. Rev. Neurol.* 14 168–181. 10.1038/nrneurol.2017.185 29377010 PMC6098968

[B11] Arotcarena M.-L. Dovero S. Prigent A. Bourdenx M. Camus S. Porras G. (2020). Bidirectional gut-to-brain and brain-to-gut propagation of synucleinopathy in non-human primates. *Brain* 143 1462–1475. 10.1093/brain/awaa096 32380543

[B12] Ashraf G. M. Tarasov V. V. Makhmutovà P. Chubarev V. N. AvilaRodriguez M. Bachurin S. O. (2019). The possibility of an infectious etiology of Alzheimer disease. *Mol. Neurobiol.* 56 4479–4491. 10.1007/s12035-018-1388-y 30338482

[B13] Bajpai P. Darra A. Agrawal A. (2018). Microbe-mitochondrion crosstalk and health: An emerging paradigm. *Mitochondrion* 39 20–25. 10.1016/j.mito.2017.08.008 28838618

[B14] Balmik A. A. Chinnathambi S. (2021). Methylation as a key regulator of Tau aggregation and neuronal health in Alzheimer’s disease. *Cell Commun. Signal* 19:51. 10.1186/s12964-021-00732-z 33962636 PMC8103764

[B15] Barbut D. Stolzenberg E. Zasloff M. (2019). Gastrointestinal immunity and alpha-synuclein. *J. Parkinson Dis.* 9 S313–S322. 10.3233/JPD-191702 31594249 PMC6839499

[B16] Bayer T. Wirths O. (2010). Intracellular accumulation of amyloid-beta - a predictor for synaptic dysfunction and neuron loss in Alzheimer’s disease. *Front. Aging Neurosci.* 2:8. 10.3389/fnagi.2010.00008 20552046 PMC2879032

[B17] Beatman E. L. Massey A. Shives K. D. Burrack K. S. Chamanian M. Morrison T. E. (2015). Alpha-synuclein expression restricts RNA viral infections in the brain. *J. Virol.* 90 2767–2782. 10.1128/JVI.02949-15 26719256 PMC4810656

[B18] Belloy M. E. Napolioni V. Greicius M. D. (2019). A quarter century of APOE and Alzheimer’s disease: progress to date and the path forward. *Neuron* 101 820–838. 10.1016/j.neuron.2019.01.056 30844401 PMC6407643

[B19] Bendor J. T. Logan T. P. Edwards R. H. (2013). The function of α-synuclein. *Neuron* 79 1044–1066. 10.1016/j.neuron.2013.09.004 24050397 PMC3866954

[B20] Berganzo K. Díez-Arrola B. Tijero B. Somme J. Lezcano E. Llorens V. (2013). Nocturnal hypertension and dysautonomia in patients with Parkinson’s disease: Are they related. *J. Neurol.* 260 1752–1756. 10.1007/s00415-013-6859-5 23412356

[B21] Bernal-Conde L. D. Ramos-Acevedo R. Reyes-Hernández M. A. Balbuena-Olvera A. J. Morales-Moreno I. D. Argüero-Sánchez R. (2020). Alpha-synuclein physiology and pathology: a perspective on cellular structures and organelles. *Front. Neurosci.* 13:1399. 10.3389/fnins.2019.01399 32038126 PMC6989544

[B22] Beydoun M. A. Beydoun H. A. Weiss J. Hossain S. El-Hajj Z. W. Zonderman A. B. (2020). *Helicobacter* pylori, periodontal pathogens, and their interactive association with incident all-cause and Alzheimer’s disease dementia in a large national survey. *Mol. Psychiatr.* 2020 1–16. 10.1038/s41380-020-0736-2 32366948

[B23] Biosa A. Outeiro T. F. Bubacco L. Bisaglia M. (2018). Diabetes mellitus as a risk factor for Parkinson’s disease: a molecular point of view. *Mol. Neurobiol.* 55 8754–8763. 10.1007/s12035-018-1025-9 29594935

[B24] Bishehsari F. Voigt R. M. Keshavarzian A. (2020). Circadian rhythms and the gut microbiota: from the metabolic syndrome to cancer. *Nat. Rev. Endocrinol.* 16 731–739. 10.1038/s41574-020-00427-4 33106657 PMC8085809

[B25] Blachier F. Mariotti F. Huneau J.-F. Tomé D. (2007). Effects of amino acid-derived luminal metabolites on the colonic epithelium and physiopathological consequences. *Amino Acids* 33 547–562. 10.1007/s00726-006-0477-9 17146590

[B26] Blanke E. N. Holmes G. M. Besecker E. M. (2021). Altered physiology of gastrointestinal vagal afferents following neurotrauma. *Neural. Regen. Res.* 16:254. 10.4103/1673-5374.290883 32859772 PMC7896240

[B27] Bollinger R. R. Barbas A. S. Bush E. L. Lin S. S. Parker W. (2007). Biofilms in the large bowel suggest an apparent function of the human vermiform appendix. *J. Theor. Biol.* 249 826–831. 10.1016/j.jtbi.2007.08.032 17936308

[B28] Bonaz B. Sinniger V. Pellissier S. (2017). The vagus nerve in the neuro-immune axis: implications in the pathology of the gastrointestinal tract. *Front. Immunol.* 8:1452. 10.3389/fimmu.2017.01452 29163522 PMC5673632

[B29] Borghammer P. (2018). How does Parkinson’s disease begin? Perspectives on neuroanatomical pathways, prions, and histology. *Mov. Disord.* 33 48–57. 10.1002/mds.27138 28843014

[B30] Borsche M. Pereira S. L. Klein C. Grünewald A. (2021). Mitochondria and Parkinson’s disease: clinical, molecular, and translational aspects. *J. Parkinson Dis.* 11 45–60. 10.3233/JPD-201981 33074190 PMC7990451

[B31] Bostock E. C. S. Kirkby K. C. Taylor B. V. M. (2017). The current status of the ketogenic diet in psychiatry. *Front. Psychiatry* 8:43. 10.3389/fpsyt.2017.00043 28373848 PMC5357645

[B32] Braak H. Tredici K. D. Rüb U. de Vos R. A. I. Jansen Steur E. N. H. Braak E. (2003). Staging of brain pathology related to sporadic Parkinson’s disease. *Neurobiol. Aging* 24 197–211. 10.1016/S0197-4580(02)00065-9 12498954

[B33] Burmester T. Weich B. Reinhardt S. Hankeln T. (2000). A vertebrate globin expressed in the brain. *Nature* 407 520–523. 10.1038/35035093 11029004

[B34] Byndloss M. X. Olsan E. E. Rivera-Chávez F. Tiffany C. R. Cevallos S. A. Lokken K. L. (2017). Microbiota-activated PPAR-γ signaling inhibits dysbiotic *Enterobacteriaceae* expansion. *Science* 357 570–575. 10.1126/science.aam9949 28798125 PMC5642957

[B35] Çamcı G. Oğuz S. (2016). Association between Parkinson’s disease and *Helicobacter* pylori. *J. Clin. Neurol.* 12 147–150. 10.3988/jcn.2016.12.2.147 26932258 PMC4828559

[B36] Canfora E. E. Jocken J. W. Blaak E. E. (2015). Short-chain fatty acids in control of body weight and insulin sensitivity. *Nat. Rev. Endocrinol.* 11 577–591. 10.1038/nrendo.2015.128 26260141

[B37] Cardoso S. M. Empadinhas N. (2018). The microbiome-mitochondria dance in prodromal Parkinson’s disease. *Front. Physiol.* 9:471. 10.3389/fphys.2018.00471 29867531 PMC5954091

[B38] Carranza-Naval M. J. Vargas-Soria M. Hierro-Bujalance C. Baena-Nieto G. Garcia-Alloza M. Infante-Garcia C. (2021). Alzheimer’s disease and diabetes: Role of diet, microbiota and inflammation in preclinical models. *Biomolecules* 11:262. 10.3390/biom11020262 33578998 PMC7916805

[B39] Chalazonitis A. Rao M. (2018). Enteric nervous system manifestations of neurodegenerative disease. *Brain Res.* 1693 207–213. 10.1016/j.brainres.2018.01.011 29360466 PMC6003851

[B40] Chen S. G. Stribinskis V. Rane M. J. Demuth D. R. Gozal E. Roberts A. M. (2016). Exposure to the functional bacterial amyloid protein curli enhances alpha-synuclein aggregation in aged Fischer 344 rats and Caenorhabditis elegans. *Sci. Rep.* 6 1–10. 10.1038/srep34477 27708338 PMC5052651

[B41] Cheng F. Vivacqua G. Yu S. (2011). The role of alpha-synuclein in neurotransmission and synaptic plasticity. *J. Chem. Neuroanat.* 42 242–248. 10.1016/j.jchemneu.2010.12.001 21167933

[B42] Cheng X. Boza-Serrano A. Turesson M. F. Deierborg T. Ekblad E. Voss U. (2016). Galectin-3 causes enteric neuronal loss in mice after left sided permanent middle cerebral artery occlusion, a model of stroke. *Sci. Rep.* 6:32893. 10.1038/srep32893 27612206 PMC5017186

[B43] Cheong J. L. Y. de Pablo-Fernandez E. Foltynie T. Noyce A. J. (2020). The association between type 2 diabetes mellitus and Parkinson’s disease. *J. Parkinson Dis.* 2020 1–15. 10.3233/JPD-191900 32333549 PMC7458510

[B44] Choi J. G. Kim N. Ju I. G. Eo H. Lim S.-M. Jang S.-E. (2018). Oral administration of *Proteus mirabilis* damages dopaminergic neurons and motor functions in mice. *Sci. Rep.* 8:1275. 10.1038/s41598-018-19646-x 29352191 PMC5775305

[B45] Choi S. H. Kim Y. H. Hebisch M. Sliwinski C. Lee S. D’Avanzo C. (2014). A three-dimensional human neural cell culture model of Alzheimer’s disease. *Nature* 515 274–278. 10.1038/nature13800 25307057 PMC4366007

[B46] Chornenkyy Y. Wang W. Wei A. Nelson P. T. (2019). Alzheimer’s disease and type 2 diabetes mellitus are distinct diseases with potential overlapping metabolic dysfunction upstream of observed cognitive decline. *Brain Pathol.* 29 3–17. 10.1111/bpa.12655 30106209 PMC6427919

[B47] Cirstea M. S. Yu A. C. Golz E. Sundvick K. Kliger D. Radisavljevic N. (2020). Microbiota composition and metabolism are associated with gut function in parkinson’s disease. *Mov. Disord.* 35 1208–1217. 10.1002/mds.28052 32357258

[B48] Citi V. Martelli A. Testai L. Marino A. Breschi M. C. Calderone V. (2014). Hydrogen sulfide releasing capacity of natural isothiocyanates: is it a reliable explanation for the multiple biological effects of Brassicaceae. *Planta Med.* 80 610–613. 10.1055/s-0034-1368591 24963613

[B49] Couto M. R. Gonçalves P. Magro F. Martel F. (2020). Microbiota-derived butyrate regulates intestinal inflammation: focus on inflammatory bowel disease. *Pharmacol. Res.* 2020:104947. 10.1016/j.phrs.2020.104947 32492488

[B50] Cryan J. F. O’Riordan K. J. Cowan C. S. M. Sandhu K. V. Bastiaanssen T. F. S. Boehme M. (2019). The microbiota-gut-brain axis. *Physiol. Rev.* 99 1877–2013. 10.1152/physrev.00018.2018 31460832

[B51] Cryan J. F. O’Riordan K. J. Sandhu K. Peterson V. Dinan T. G. (2020). The gut microbiome in neurological disorders. *Lancet Neurol.* 19 179–194. 10.1016/S1474-4422(19)30356-4 31753762

[B52] Cui P. Ma X. Li H. Lang W. Hao J. (2018). Shared biological pathways between Alzheimer’s disease and ischemic stroke. *Front. Neurosci.* 12:605. 10.3389/fnins.2018.00605 30245614 PMC6137293

[B53] Cunnane S. C. Trushina E. Morland C. Prigione A. Casadesus G. Andrews Z. B. (2020). Brain energy rescue: an emerging therapeutic concept for neurodegenerative disorders of ageing. *Nat. Rev. Drug Discov.* 19 609–633. 10.1038/s41573-020-0072-x 32709961 PMC7948516

[B54] Czepiel J. Dróżdż M. Pituch H. Kuijper E. J. Perucki W. Mielimonka A. (2019). Clostridium difficile infection: review. *Eur. J. Clin. Microbiol*. 38, 1211–1221. 10.1007/s10096-019-03539-6 30945014 PMC6570665

[B55] D’Andrea M. R. (2016). *Intracellular Consequences of Amyloid in Alzheimer’s Disease.* Amsterdam: Elsevier.

[B56] D’Andrea M. R. Nagele R. G. (2010). Morphologically distinct types of amyloid plaques point the way to a better understanding of Alzheimer’s disease pathogenesis. *Biotech. Histochem.* 85 133–147. 10.3109/10520290903389445 20121465

[B57] D’Argenio V. Sarnataro D. (2019). Microbiome influence in the pathogenesis of prion and Alzheimer’s diseases. *Int. J. Mol. Sci.* 20:4704. 10.3390/ijms20194704 31547531 PMC6801937

[B58] Del Tredici K. Braak H. (2020). To stage, or not to stage. *Curr. Opin. Neurobiol.* 61 10–22. 10.1016/j.conb.2019.11.008 31862625

[B59] Desmet A.-S. Cirillo C. Tack J. Vandenberghe W. Vanden Berghe P. (2017). Live calcium and mitochondrial imaging in the enteric nervous system of Parkinson patients and controls. *eLife* 6:e26850. 10.7554/eLife.26850 28825895 PMC5565316

[B60] Dickson D. W. Braak H. Duda J. E. Duyckaerts C. Gasser T. Halliday G. M. (2009). Neuropathological assessment of Parkinson’s disease: refining the diagnostic criteria. *Lancet Neurol.* 8 1150–1157. 10.1016/S1474-4422(09)70238-8 19909913

[B61] Divyashri G. Krishna G. Muralidhara Prapulla S. G. (2015). Probiotic attributes, antioxidant, anti-inflammatory and neuromodulatory effects of Enterococcus faecium CFR 3003: in vitro and in vivo evidence. *J. Med. Microbiol.* 64 1527–1540. 10.1099/jmm.0.000184 26450608

[B62] Donohoe D. R. Garge N. Zhang X. Sun W. O’Connell T. M. Bunger M. K. (2011). The microbiome and butyrate regulate energy metabolism and autophagy in the mammalian colon. *Cell Metab.* 13 517–526. 10.1016/j.cmet.2011.02.018 21531334 PMC3099420

[B63] Dorsey E. R. Bloem B. R. (2018). The parkinson pandemic – a call to action. *JAMA Neurol.* 75 9–10. 10.1001/jamaneurol.2017.3299 29131880

[B64] Dorsey E. R. Constantinescu R. Thompson J. P. Biglan K. M. Holloway R. G. Kieburtz K. (2007). Projected number of people with Parkinson disease in the most populous nations, 2005 through 2030. *Neurology* 68 384–386. 10.1212/01.wnl.0000247740.47667.03 17082464

[B65] Doulberis M. Kotronis G. Thomann R. Polyzos S. A. Boziki M. Gialamprinou D. (2018). Impact of *Helicobacter* pylori on Alzheimer’s disease: what do we know so far. *Helicobacter* 23 e12454. 10.1111/hel.12454 29181894

[B66] Duncan S. H. Flint H. J. (2013). Probiotics and prebiotics and health in ageing populations. *Maturitas* 75 44–50. 10.1016/j.maturitas.2013.02.004 23489554

[B67] Fani L. Wolters F. J. Ikram M. K. Bruno M. J. Hofman A. Koudstaal P. J. (2018). *Helicobacter* pylori and the risk of dementia: a population-based study. *Alzheimers Dement* 14 1377–1382. 10.1016/j.jalz.2018.05.005 29935141

[B68] Fasano A. Bove F. Gabrielli M. Petracca M. Zocco M. A. Ragazzoni E. (2013). The role of small intestinal bacterial overgrowth in Parkinson’s disease. *Mov. Disord.* 28 1241–1249. 10.1002/mds.25522 23712625

[B69] Feng S.-T. Wang Z.-Z. Yuan Y.-H. Sun H.-M. Chen N.-H. Zhang Y. (2021). Update on the association between alpha-synuclein and tau with mitochondrial dysfunction: Implications for Parkinson’s disease. *Eur. J. Neurosci.* 53 2946–2959. 10.1111/ejn.14699 32031280

[B70] Fernández J. Redondo-Blanco S. Gutiérrez-del-Río I. Miguélez E. M. Villar C. J. Lombo F. (2016). Colon microbiota fermentation of dietary prebiotics towards short-chain fatty acids and their roles as anti-inflammatory and antitumour agents: a review. *J. Funct. Foods* 25 511–522. 10.1016/j.jff.2016.06.032

[B71] Filippini A. Gennarelli M. Russo I. (2019). α-Synuclein and glia in parkinson’s disease: a beneficial or a detrimental duet for the endo-lysosomal system. *Cell Mol. Neurobiol.* 39 161–168. 10.1007/s10571-019-00649-9 30637614 PMC11469870

[B72] Fitzgerald E. Murphy S. Martinson H. A. (2019). Alpha-synuclein pathology and the role of the microbiota in Parkinson’s disease. *Front. Neurosci.* 13:369. 10.3389/fnins.2019.00369 31068777 PMC6491838

[B73] Floud S. Simpson R. F. Balkwill A. Brown A. Goodill A. Gallacher J. (2020). Body mass index, diet, physical inactivity, and the incidence of dementia in 1 million UK women. *Neurology* 94 e123–e132. 10.1212/WNL.0000000000008779 31852815 PMC6988985

[B74] Foo J. H. Culvenor J. G. Ferrero R. L. Kwok T. Lithgow T. Gabriel K. (2010). Both the p33 and p55 subunits of the *Helicobacter* pylori VacA toxin are targeted to mammalian mitochondria. *J. Mol. Biol.* 401 792–798. 10.1016/j.jmb.2010.06.065 20615415

[B75] Forster M. J. Morris P. Sohal R. S. (2003). Genotype and age influence the effect of caloric intake on mortality in mice. *FASEB J.* 17 690–692. 10.1096/fj.02-0533fje 12586746 PMC2839882

[B76] Friedland R. P. Chapman M. R. (2017). The role of microbial amyloid in neurodegeneration. *PLoS Pathog.* 13:e1006654. 10.1371/journal.ppat.1006654 29267402 PMC5739464

[B77] Fu M. Zhang W. Wu L. Yang G. Li H. Wang R. (2012). Hydrogen sulfide (H2S) metabolism in mitochondria and its regulatory role in energy production. *P Natl. Acad. Sci. USA* 109:2943. 10.1073/pnas.1115634109 22323590 PMC3287003

[B78] Fülling C. Dinan T. G. Cryan J. F. (2019). Gut Microbe to Brain Signaling: What Happens in Vagus. *Neuron* 101 998–1002. 10.1016/j.neuron.2019.02.008 30897366

[B79] Fulop T. Witkowski J. M. Bourgade K. Khalil A. Zerif E. Larbi A. (2018). Can an infection hypothesis explain the beta amyloid hypothesis of Alzheimer’s Disease. *Front. Aging Neurosci.* 10:224. 10.3389/fnagi.2018.00224 30087609 PMC6066504

[B80] Furness J. B. (2012). The enteric nervous system and neurogastroenterology. *Nat. Rev. Gastro. Hepat.* 9 286–294. 10.1038/nrgastro.2012.32 22392290

[B81] GBD 2015 Disease and Injury Incidence and Prevalence Collaborators (2016). Global, regional, and national incidence, prevalence, and years lived with disability for 310 diseases and injuries, 1990–2015: a systematic analysis for the Global Burden of Disease Study 2015. *Lancet* 388 1545–1602. 10.1016/s0140-6736(16)31678-6 27733282 PMC5055577

[B82] Gill S. K. Rossi M. Bajka B. Whelan K. (2021). Dietary fibre in gastrointestinal health and disease. *Nat. Rev. Gastro. Hepat.* 18 101–116. 10.1038/s41575-020-00375-4 33208922

[B83] Giovinazzo D. Bursac B. Sbodio J. I. Nalluru S. Vignane T. Snowman A. M. (2021). Hydrogen sulfide is neuroprotective in Alzheimer’s disease by sulfhydrating GSK3β and inhibiting Tau hyperphosphorylation. *P. Natl. Acad. Sci. USA* 118:e2017225118. 10.1073/pnas.2017225118 33431651 PMC7848711

[B84] Goedert M. (2015). Alzheimer’s and Parkinson’s diseases: the prion concept in relation to assembled Aβ, tau, and α-synuclein. *Science* 349:1255555. 10.1126/science.1255555 26250687

[B85] Golovko M. Y. Barceló-Coblijn G. Castagnet P. I. Austin S. Combs C. K. Murphy E. J. (2009). The role of α-synuclein in brain lipid metabolism: a downstream impact on brain inflammatory response. *Mol. Cell Biochem.* 326 55–66. 10.1007/s11010-008-0008-y 19116775

[B86] Gonzaìlez-Rodriìguez P. Zampese E. Stout K. A. Guzman J. N. Ilijic E. Yang B. (2021). Disruption of mitochondrial complex I induces progressive parkinsonism. *Nature* 599 650–656. 10.1038/s41586-021-04059-0 34732887 PMC9189968

[B87] Goubern M. Andriamihaja M. Nübel T. Blachier F. Bouillaud F. (2007). Sulfide, the first inorganic substrate for human cells. *FASEB J.* 21 1699–1706. 10.1096/fj.06-7407com 17314140

[B88] Greco T. Glenn T. C. Hovda D. A. Prins M. L. (2016). Ketogenic diet decreases oxidative stress and improves mitochondrial respiratory complex activity. *J. Cerebr. Blood F Met.* 36 1603–1613. 10.1177/0271678X15610584 26661201 PMC5012517

[B89] Green R. C. Schneider L. S. Amato D. A. Beelen A. P. Wilcock G. Swabb E. A. (2009). Effect of tarenflurbil on cognitive decline and activities of daily living in patients with mild alzheimer disease: a randomized controlled trial. *J. Amer. Med. Assoc.* 302 2557–2564. 10.1001/jama.2009.1866 20009055 PMC2902875

[B90] Groschwitz K. R. Hogan S. P. (2009). Intestinal barrier function: molecular regulation and disease pathogenesis. *J. Allergy Clin. Immun.* 124 3–22. 10.1016/j.jaci.2009.05.038 19560575 PMC4266989

[B91] Guo J. L. Covell D. J. Daniels J. P. Iba M. Stieber A. Zhang B. (2013). Distinct α-synuclein strains differentially promote tau inclusions in neurons. *Cell* 154 103–117. 10.1016/j.cell.2013.05.057 23827677 PMC3820001

[B92] Guo T. Noble W. Hanger D. P. (2017). Roles of tau protein in health and disease. *Acta Neuropathol.* 133 665–704. 10.1007/s00401-017-1707-9 28386764 PMC5390006

[B93] Hampe C. S. Roth C. L. (2017). Probiotic strains and mechanistic insights for the treatment of type 2 diabetes. *Endocrine* 58 207–227. 10.1007/s12020-017-1433-z 29052181

[B94] Harach T. Marungruang N. Duthilleul N. Cheatham V. Mc Coy K. D. Frisoni G. (2017). Reduction of Abeta amyloid pathology in APPPS1 transgenic mice in the absence of gut microbiota. *Sci. Rep.* 7:41802. 10.1038/srep41802 28176819 PMC5297247

[B95] Haran J. P. McCormick B. A. (2021). Aging, frailty, and the microbiome—how dysbiosis influences human aging and disease. *Gastroenterology* 160 507–523. 10.1053/j.gastro.2020.09.060 33307030 PMC7856216

[B96] Helander H. F. Fändriks L. (2014). Surface area of the digestive tract–revisited. *Scand. J. Gastroentero.* 49 681–689. 10.3109/00365521.2014.898326 24694282

[B97] Hirose M. Künstner A. Schilf P. Sünderhauf A. Rupp J. Jöhren O. (2017). Mitochondrial gene polymorphism is associated with gut microbial communities in mice. *Sci. Rep.* 7:15293. 10.1038/s41598-017-15377-7 29127319 PMC5681637

[B98] Ho L. Ono K. Tsuji M. Mazzola P. Singh R. Pasinetti G. M. (2018). Protective roles of intestinal microbiota derived short chain fatty acids in Alzheimer’s disease-type beta-amyloid neuropathological mechanisms. *Expert Rev. Neurother.* 18 83–90. 10.1080/14737175.2018.1400909 29095058 PMC5958896

[B99] Hohman T. J. Dumitrescu L. Barnes L. L. Thambisetty M. Beecham G. Kunkle B. (2018). Sex-specific association of apolipoprotein E with cerebrospinal fluid levels of tau. *JAMA Neurol.* 75 989–998. 10.1001/jamaneurol.2018.0821 29801024 PMC6142927

[B100] Holmqvist S. Chutna O. Bousset L. Aldrin-Kirk P. Li W. Björklund T. (2014). Direct evidence of Parkinson pathology spread from the gastrointestinal tract to the brain in rats. *Acta Neuropathol.* 128 805–820. 10.1007/s00401-014-1343-6 25296989

[B101] Hou X. Yuan Y. Sheng Y. Yuan B. Wang Y. Zheng J. (2017). GYY4137, an H2S slow-releasing donor, prevents nitrative stress and α-synuclein nitration in an MPTP mouse model of Parkinson’s disease. *Front. Pharmacol.* 8:741. 10.3389/fphar.2017.00741 29163149 PMC5671206

[B102] Hou Y. Dan X. Babbar M. Wei Y. Hasselbalch S. G. Croteau D. L. (2019). Ageing as a risk factor for neurodegenerative disease. *Nat. Rev. Neurol.* 15 565–581. 10.1038/s41582-019-0244-7 31501588

[B103] Huang H.-K. Wang J.-H. Lei W.-Y. Chen C.-L. Chang C.-Y. Liou L.-S. (2018). *Helicobacter pylori* infection is associated with an increased risk of Parkinson’s disease: a population-based retrospective cohort study. *Parkinsonism Rel. D* 47 26–31. 10.1016/j.parkreldis.2017.11.331 29174171

[B104] Huang Y. Liao J. Liu X. Zhong Y. Cai X. Long L. (2021). Review: the role of intestinal dysbiosis in parkinson’s disease. *Front. Cell Infect. Microbiol.* 11:615075. 10.3389/fcimb.2021.615075 33968794 PMC8100321

[B105] Huang Z. Yan Q. Wang Y. Zou Q. Li J. Liu Z. (2020). Role of mitochondrial dysfunction in the pathology of amyloid-β. *J. Alzheimers Dis.* 2020 1–10. 10.1038/s41598-020-68882-7 33044180

[B106] Hugon P. Dufour J.-C. Colson P. Fournier P.-E. Sallah K. Raoult D. (2015). A comprehensive repertoire of prokaryotic species identified in human beings. *Lancet Infect. Dis.* 15 1211–1219. 10.1016/S1473-3099(15)00293-5 26311042

[B107] Huynh T.-P. V. Davis A. A. Ulrich J. D. Holtzman D. M. (2017). Apolipoprotein E and Alzheimer’s disease: the influence of apolipoprotein E on amyloid-β and other amyloidogenic proteins. *J. Lipid. Res.* 58 824–836. 10.1194/jlr.R075481 28246336 PMC5408619

[B108] Imbimbo B. P. (2009). Why did tarenflurbil fail in alzheimer’s disease. *J. Alzheimers Dis.* 17 757–760. 10.3233/JAD-2009-1092 19542625

[B109] Jackson D. N. Theiss A. L. (2020). Gut bacteria signaling to mitochondria in intestinal inflammation and cancer. *Gut. Microbes.* 11 285–304. 10.1080/19490976.2019.1592421 30913966 PMC7524274

[B110] Janke R. Dodson A. E. Rine J. (2015). Metabolism and epigenetics. *Annu. Rev. Cell Dev. Biol.* 31 473–496. 10.1146/annurev-cellbio-100814-125544 26359776 PMC5091661

[B111] Jankovska N. Olejar T. Matej R. (2021). Extracellular amyloid deposits in alzheimer’s and creutzfeldt–jakob disease: similar behavior of different proteins. *Int. J. Mol. Sci.* 22:7. 10.3390/ijms22010007 33374972 PMC7792617

[B112] Johnston I. G. Williams B. P. (2016). Evolutionary inference across eukaryotes identifies specific pressures favoring mitochondrial gene retention. *Cell Syst.* 2 101–111. 10.1016/j.cels.2016.01.013 27135164

[B113] Kalia L. V. Lang A. E. (2015). Parkinson’s disease. *Lancet* 386 896–912. 10.1016/S0140-6736(14)61393-3 25904081

[B114] Kamath A. F. Chauhan A. K. Kisucka J. Dole V. S. Loscalzo J. Handy D. E. (2006). Elevated levels of homocysteine compromise blood-brain barrier integrity in mice. *Blood* 107 591–593. 10.1182/blood-2005-06-2506 16189268 PMC1895614

[B115] Kellingray L. Tapp H. S. Saha S. Doleman J. F. Narbad A. Mithen R. F. (2017). Consumption of a diet rich in Brassica vegetables is associated with a reduced abundance of sulphate-reducing bacteria: a randomised crossover study. *Mol. Nutr. Food Res.* 61:1600992. 10.1002/mnfr.201600992 28296348 PMC5600105

[B116] Killinger B. A. Madaj Z. Sikora J. W. Rey N. Haas A. J. Vepa Y. (2018). The vermiform appendix impacts the risk of developing Parkinson’s disease. *Sci. Transl. Med.* 10:eaar5280. 10.1126/scitranslmed.aar5280 30381408 PMC6319259

[B117] Kim J. Guan K.-L. (2019). mTOR as a central hub of nutrient signalling and cell growth. *Nat. Cell Biol.* 21 63–71. 10.1038/s41556-018-0205-1 30602761

[B118] Kim R. Jun J. S. (2020). Impact of overweight and obesity on functional and clinical outcomes of early parkinson’s disease. *J. Am. Med. Dir. Assoc.* 21 697–700. 10.1016/j.jamda.2019.11.019 31928933

[B119] Kim S. Kwon S.-H. Kam T.-I. Panicker N. Karuppagounder S. S. Lee S. (2019). Transneuronal propagation of pathologic α-synuclein from the gut to the brain models Parkinson’s disease. *Neuron* 103 627–641. 10.1016/j.neuron.2019.05.035 31255487 PMC6706297

[B120] Kitada M. Ogura Y. Monno I. Xu J. Koya D. (2021). Effect of methionine restriction on aging: its relationship to oxidative stress. *Biomedicines* 9:130. 10.3390/biomedicines9020130 33572965 PMC7911310

[B121] Kitamoto S. Alteri C. J. Rodrigues M. Nagao-Kitamoto H. Sugihara K. Himpsl S. D. (2020). Dietary L-serine confers a competitive fitness advantage to *Enterobacteriaceae* in the inflamed gut. *Nat. Microbiol.* 5 116–125. 10.1038/s41564-019-0591-6 31686025 PMC6925351

[B122] Knudsen K. E. B. Lærke H. N. Hedemann M. S. Nielsen T. S. Ingerslev A. K. Gundelund Nielsen D. S. (2018). Impact of diet-modulated butyrate production on intestinal barrier function and inflammation. *Nutrients* 10:1499. 10.3390/nu10101499 30322146 PMC6213552

[B123] Konjar S. Frising U. C. Ferreira C. Hinterleitner R. Mayassi T. Zhang Q. (2018). Mitochondria maintain controlled activation state of epithelial-resident T lymphocytes. *Sci. Immunol.* 3:eaan2543. 10.1126/sciimmunol.aan2543 29934344 PMC6690060

[B124] Kowalski K. Mulak A. (2019). Brain-gut-microbiota axis in Alzheimer’s disease. *J. Neurogastroenterol.* 25 48–60. 10.5056/jnm18087 30646475 PMC6326209

[B125] Kramer P. Bressan P. (2015). Humans as superorganisms: how microbes, viruses, imprinted genes, and other selfish entities shape our behavior. *Perspect. Psychol. Sci.* 10 464–481. 10.1177/1745691615583131 26177948

[B126] Kramer P. Bressan P. (2018). Our (mother’s) mitochondria and our mind. *Perspect. Psychol. Sci.* 13 88–100. 10.1177/1745691617718356 28937858 PMC5761714

[B127] Kramer P. Bressan P. (2019). Mitochondria inspire a lifestyle. *Adv. Anat. Embryol. Cell Biol.* 231 105–126. 10.1007/102_2018_5 30610376

[B128] Kramer P. Bressan P. (2021). Infection threat shapes our social instincts. *Behav. Ecol. Sociobiol.* 75 1–18. 10.1007/s00265-021-02975-9 33583997 PMC7873116

[B129] Kumar D. K. V. Choi S. H. Washicosky K. J. Eimer W. A. Tucker S. Ghofrani J. (2016). Amyloid-β peptide protects against microbial infection in mouse and worm models of Alzheimer’s disease. *Sci. Transl. Med.* 8:340ra72. 10.1126/scitranslmed.aaf1059 27225182 PMC5505565

[B130] Lane N. (2015). *The Vital Question: Energy, Evolution, and the Origins of Complex Life.* London: WW Norton & Company.

[B131] Lane N. Martin W. (2010). The energetics of genome complexity. *Nature* 467 929–934. 10.1038/nature09486 20962839

[B132] Lautenschäger J. Schierle G. S. K. (2019). Mitochondrial degradation of amyloidogenic proteins—A new perspective for neurodegenerative diseases. *Prog. Neurobiol.* 181:101660. 10.1016/j.pneurobio.2019.101660 31301323

[B133] Lautenschäger J. Wagner-Valladolid S. Stephens A. D. Fernández-Villegas A. Hockings C. Mishra A. (2020). Intramitochondrial proteostasis is directly coupled to α-synuclein and amyloid β1-42 pathologies. *J. Biol. Chem.* 295 10138–10152. 10.1074/jbc.RA119.011650 32385113 PMC7383368

[B134] Lazic D. Tesic V. Jovanovic M. Brkic M. Milanovic D. Zlokovic B. V. (2020). Every-other-day feeding exacerbates inflammation and neuronal deficits in 5XFAD mouse model of Alzheimer’s disease. *Neurobiol. Dis.* 136:104745. 10.1016/j.nbd.2020.104745 31931140

[B135] Le T. N. Chiu C.-H. Hsieh P.-C. (2020). Bioactive compounds and bioactivities of brassica oleracea l. var. italica sprouts and microgreens: An updated overview from a nutraceutical perspective. *Plants* 9:946. 10.3390/plants9080946 32727144 PMC7465980

[B136] LeBlanc J. G. Chain F. Martín R. Bermúdez-Humarán L. G. Courau S. Langella P. (2017). Beneficial effects on host energy metabolism of short-chain fatty acids and vitamins produced by commensal and probiotic bacteria. *Microb. Cell Fact.* 16:79. 10.1186/s12934-017-0691-z 28482838 PMC5423028

[B137] Lewis K. Lutgendorff F. Phan V. Söderholm J. D. Sherman P. M. McKay D. M. (2010). Enhanced translocation of bacteria across metabolically stressed epithelia is reduced by butyrate. *Inflamm. Bowel. Dis.* 16 1138–1148. 10.1002/ibd.21177 20024905

[B138] Li F. Hearn M. Bennett L. E. (2021). The role of microbial infection in the pathogenesis of Alzheimer’s disease and the opportunity for protection by anti-microbial peptides. *Crit. Rev. Microbiol.* 47 240–253. 10.1080/1040841X.2021.1876630 33555958

[B139] Li H. M. Liu C. C. Zheng H. Huang T. Y. (2018). Amyloid, tau, pathogen infection and antimicrobial protection in Alzheimer’s disease–conformist, nonconformist, and realistic prospects for AD pathogenesis. *Transl. Neurodegen.* 7:34. 10.1186/s40035-018-0139-3 30603085 PMC6306008

[B140] Liddle R. A. (2018). Parkinson’s disease from the gut. *Brain Res.* 1693 201–206. 10.1016/j.brainres.2018.01.010 29360467 PMC6003841

[B141] Lilamand M. Porte B. Cognat E. Hugon J. Mouton-Liger F. Paquet C. (2020). Are ketogenic diets promising for Alzheimer’s disease? a translational review. *Alzheimers Res. Ther.* 12 1–10. 10.1186/s13195-020-00615-4 32290868 PMC7158135

[B142] Lin H.-C. Hsieh H.-M. Chen Y.-H. Hu M.-L. (2009). S-Adenosylhomocysteine increases β-amyloid formation in BV-2 microglial cells by increased expressions of β-amyloid precursor protein and presenilin 1 and by hypomethylation of these gene promoters. *NeuroToxicology* 30 622–627. 10.1016/j.neuro.2009.03.011 19635394

[B143] Lionnet A. Leclair-Visonneau L. Neunlist M. Murayama S. Takao M. Adler C. H. (2018). Does Parkinson’s disease start in the gut. *Acta Neuropathol.* 135 1–12. 10.1007/s00401-017-1777-8 29039141

[B144] Litvak Y. Byndloss M. X. Tsolis R. M. Bäumler A. J. (2017). Dysbiotic *Proteobacteria* expansion: a microbial signature of epithelial dysfunction. *Curr. Opin. Microbiol.* 39 1–6. 10.1016/j.mib.2017.07.003 28783509

[B145] Litvinova L. Atochin D. N. Fattakhov N. Vasilenko M. Zatolokin P. Kirienkova E. (2015). Nitric oxide and mitochondria in metabolic syndrome. *Front. Physiol.* 6:20. 10.3389/fphys.2015.00020 25741283 PMC4330700

[B146] Liu Y. Niu L. Liu X. Cheng C. Le W. (2021). Recent progress in non-motor features of parkinson’s disease with a focus on circadian rhythm dysregulation. *Neurosci. Bull.* 2021 1–15. 10.1007/s12264-021-00711-x 34128188 PMC8275711

[B147] Liu Y. Xue L. Zhang Y. Xie A. (2020). Association between stroke and parkinson’s disease: a meta-analysis. *J. Mol. Neurosci.* 70 1169–1176. 10.1007/s12031-020-01524-9 32180111

[B148] Loenen W. A. (2006). S-Adenosylmethionine: jack of all trades and master of everything? *Biochem. Soc. T* 34 330–333. 10.1042/BST20060330 16545107

[B149] Lohmann S. Bernis M. E. Tachu B. J. Ziemski A. Grigoletto J. Tamgüney G. (2019). Oral and intravenous transmission of α-synuclein fibrils to mice. *Acta Neuropathol.* 138 515–533. 10.1007/s00401-019-02037-5 31230104 PMC6778172

[B150] Lopes A. F. C. (2020). Mitochondrial metabolism and DNA methylation: a review of the interaction between two genomes. *Clin. Epigen.* 12:182. 10.1186/s13148-020-00976-5 33228792 PMC7684747

[B151] López-Armada M. J. Riveiro-Naveira R. R. Vaamonde-García C. Valcárcel-Ares M. N. (2013). Mitochondrial dysfunction and the inflammatory response. *Mitochondrion* 13 106–118. 10.1016/j.mito.2013.01.003 23333405

[B152] Lu K. Nicholas J. M. Pertzov Y. Grogan J. Husain M. Pavisic I. M. (2021). Dissociable effects of APOE ε4 and β-amyloid pathology on visual working memory. *Nat. Aging* 1 1002–1009. 10.1038/s43587-021-00117-4 34806027 PMC7612005

[B153] Lubomski M. Tan A. H. Lim S.-Y. Holmes A. J. Davis R. L. Sue C. M. (2019). Parkinson’s disease and the gastrointestinal microbiome. *J. Neurol.* 2019 1–17. 10.1007/s00415-019-09320-1 31041582

[B154] Luca M. Di Mauro M. Di Mauro M. Luca A. (2019). Gut microbiota in alzheimer’s disease, depression, and type 2 diabetes mellitus: the role of oxidative stress. *Oxid. Med. Cell Longev.* 2019:4730539. 10.1155/2019/4730539 31178961 PMC6501164

[B155] Lundmark K. Westermark G. T. Olsén A. Westermark P. (2005). Protein fibrils in nature can enhance amyloid protein A amyloidosis in mice: Cross-seeding as a disease mechanism. *P. Natl. Acad. Sci. USA* 102 6098–6102. 10.1073/pnas.0501814102 15829582 PMC1087940

[B156] Ma J. Coarfa C. Qin X. Bonnen P. E. Milosavljevic A. Versalovic J. (2014). mtDNA haplogroup and single nucleotide polymorphisms structure human microbiome communities. *BMC Genomics* 15:257. 10.1186/1471-2164-15-257 24694284 PMC4234434

[B157] Ma N. Ma X. (2019). Dietary amino acids and the gut-microbiome-immune axis: physiological metabolism and therapeutic prospects. *Compr. Rev. Food Sci. F* 18 221–242. 10.1111/1541-4337.12401 33337014

[B158] Makin S. (2018). The amyloid hypothesis on trial. *Nature* 559 S4–S4. 10.1038/d41586-018-05719-4 30046080

[B159] Mallikarjuna N. Praveen K. Yellamma K. (2016). Role of lactobacillus plantarum MTCC1325 in membrane-bound transport ATPases system in Alzheimer’s disease-induced rat brain. *Bioimpacts* 6 203–209. 10.15171/bi.2016.27 28265536 PMC5326668

[B160] Maniar K. Moideen A. Mittal A. Patil A. Chakrabarti A. Banerjee D. (2017). A story of metformin-butyrate synergism to control various pathological conditions as a consequence of gut microbiome modification: genesis of a wonder drug. *Pharmacol. Res.* 117 103–128. 10.1016/j.phrs.2016.12.003 27939359

[B161] Marx W. Scholey A. Firth J. D’Cunha N. M. Lane M. Hockey M. (2020). Prebiotics, probiotics, fermented foods and cognitive outcomes: A meta-analysis of randomized controlled trials. *Neurosci. Biobehav. R* 118 472–484. 10.1016/j.neubiorev.2020.07.036 32860802

[B162] Mattson M. P. Arumugam T. V. (2018). Hallmarks of brain aging: adaptive and pathological modification by metabolic states. *Cell metab.* 27 1176–1199. 10.1016/j.cmet.2018.05.011 29874566 PMC6039826

[B163] Mattson M. P. Moehl K. Ghena N. Schmaedick M. Cheng A. (2018). Intermittent metabolic switching, neuroplasticity and brain health. *Nat. Rev. Neurosci.* 19:63. 10.1038/nrn.2017.156 29321682 PMC5913738

[B164] McCarty M. F. Lerner A. (2021). Perspective: low risk of parkinson’s disease in quasi-vegan cultures may reflect GCN2-mediated upregulation of parkin. *Adv. Nutr.* 12 355–362. 10.1093/advances/nmaa112 32945884 PMC8009740

[B165] Miller A. L. Bessho S. Grando K. Tukel C. (2021). Microbiome or infections: amyloid-containing biofilms as a trigger for complex human diseases. *Front. Immunol.* 12:514. 10.3389/fimmu.2021.638867 33717189 PMC7952436

[B166] Minter M. R. Hinterleitner R. Meisel M. Zhang C. Leone V. Zhang X. (2017). Antibiotic-induced perturbations in microbial diversity during post-natal development alters amyloid pathology in an aged APPSWE/PS1ΔE9 murine model of Alzheimer’s disease. *Sci. Rep.* 7:10411. 10.1038/s41598-017-11047-w 28874832 PMC5585265

[B167] Miraglia F. Colla E. (2019). Microbiome, parkinson’s disease and molecular mimicry. *Cells* 8:222. 10.3390/cells8030222 30866550 PMC6468760

[B168] Moir R. D. Lathe R. Tanzi R. E. (2018). The antimicrobial protection hypothesis of Alzheimer’s disease. *Alzheimers Dement* 14 1602–1614. 10.1016/j.jalz.2018.06.3040 30314800

[B169] Moreno-Loshuertos R. Acin-Perez R. Fernandez-Silva P. Movilla N. Perez-Martos A. de Cordoba S. R. (2006). Differences in reactive oxygen species production explain the phenotypes associated with common mouse mitochondrial DNA variants. *Nat. Genet.* 38 1261–1268. 10.1038/ng1897 17013393

[B170] Moreno-Loshuertos R. Perez Martos A. Fernandez Silva P. Enriquez J. A. (2013). Length variation in the mouse mitochondrial tRNAA rg DHU loop size promotes oxidative phosphorylation functional differences. *FEBS J.* 280 4983–4998. 10.1111/febs.12466 23910637

[B171] Mottawea W. Chiang C.-K. Muhlbauer M. Starr A. E. Butcher J. Abujamel T. (2016). Altered intestinal microbiota–host mitochondria crosstalk in new onset Crohn’s disease. *Nat. Commun.* 7:13419. 10.1038/ncomms13419 27876802 PMC5122959

[B172] Mulvey L. Sinclair A. Selman C. (2014). Lifespan modulation in mice and the confounding effects of genetic background. *J. Genet. Genomics* 41 497–503. 10.1016/j.jgg.2014.06.002 25269675 PMC4257991

[B173] Munoz-Pinto M. F. Empadinhas N. Cardoso S. M. (2021). The neuromicrobiology of Parkinson’s disease: a unifying theory. *Ageing Res. Rev.* 70:101396. 10.1016/j.arr.2021.101396 34171417

[B174] Nair A. T. Ramachandran V. Joghee N. M. Antony S. Ramalingam G. (2018). Gut microbiota dysfunction as reliable non-invasive early diagnostic biomarkers in the pathophysiology of Parkinson’s disease: a critical review. *J. Neurogastroenterol.* 24:30. 10.5056/jnm17105 29291606 PMC5753901

[B175] Nguyen P. H. Ramamoorthy A. Sahoo B. R. Zheng J. Faller P. Straub J. E. (2021). Amyloid oligomers: a joint experimental/computational perspective on Alzheimer’s disease, Parkinson’s disease, Type II diabetes, and amyotrophic lateral sclerosis. *Chem. Rev.* 121 2545–2647. 10.1021/acs.chemrev.0c01122 33543942 PMC8836097

[B176] Niesler B. Kuerten S. Demir I. E. Schäfer K.-H. (2021). Disorders of the enteric nervous system—a holistic view. *Nat. Rev. Gastro. Hepat.* 18 393–410. 10.1038/s41575-020-00385-2 33514916

[B177] Notkola I. L. Sulkava R. Pekkanen J. Erkinjuntti T. Ehnholm C. Kivinen P. (1998). Serum total cholesterol, apolipoprotein E (Notkola et al., 1998) e4 allele, and Alzheimer’s disease. *Neuroepidemiology* 17 14–20. 10.1159/000026149 9549720

[B178] Nurrahma B. A. Tsao S.-P. Wu C.-H. Yeh T.-H. Hsieh P.-S. Panunggal B. (2021). Probiotic supplementation facilitates recovery of 6-OHDA-induced motor deficit via improving mitochondrial function and energy metabolism. *Front. Aging Neurosci.* 13:230. 10.3389/fnagi.2021.668775 34025392 PMC8137830

[B179] Pacelli C. Giguere N. Bourque M. J. Levesque M. Slack R. S. Trudeau L.-E. (2015). Elevated mitochondrial bioenergetics and axonal arborization size are key contributors to the vulnerability of dopamine neurons. *Curr. Biol.* 25 2349–2360. 10.1016/j.cub.2015.07.050 26320949

[B180] Pal V. K. Bandyopadhyay P. Singh A. (2018). Hydrogen sulfide in physiology and pathogenesis of bacteria and viruses. *IUBMB Life* 70 393–410. 10.1002/iub.1740 29601123 PMC6029659

[B181] Pan L. Meng L. He M. Zhang Z. (2021). Tau in the pathophysiology of parkinson’s disease. *J. Mol. Neurosci.* 2021 1–13. 10.1007/s12031-020-01776-5 33459970 PMC8585831

[B182] Pang S. Li J. Zhang Y. Chen J. (2018). Meta-analysis of the relationship between the apoe gene and the onset of parkinson’s disease dementia. *Parkinsons Dis.* 2018:9497147. 10.1155/2018/9497147 30405900 PMC6204165

[B183] Panza F. Lozupone M. Logroscino G. Imbimbo B. P. (2019). A critical appraisal of amyloid-β-targeting therapies for Alzheimer disease. *Nat. Rev. Neurol.* 15 73–88. 10.1038/s41582-018-0116-6 30610216

[B184] Parihar M. S. Brewer G. J. (2010). Amyloid-β as a modulator of synaptic plasticity. *J. Alzheimers Dis.* 22 741–763. 10.3233/JAD-2010-101020 20847424 PMC3079354

[B185] Park S.-C. Moon J. C. Shin S. Y. Son H. Jung Y. J. Kim N.-H. (2016). Functional characterization of alpha-synuclein protein with antimicrobial activity. *Biochem. Bioph. Res. Co.* 478 924–928. 10.1016/j.bbrc.2016.08.052 27520375

[B186] Paul B. D. (2021). Neuroprotective roles of the reverse transsulfuration pathway in alzheimer’s disease. *Front. Aging Neurosci.* 13:659402. 10.3389/fnagi.2021.659402 33796019 PMC8007787

[B187] Paul B. D. Snyder S. H. (2018). Gasotransmitter hydrogen sulfide signaling in neuronal health and disease. *Biochem. Pharmacol.* 149 101–109. 10.1016/j.bcp.2017.11.019 29203369 PMC5868969

[B188] Peng X. Meng J. Chi T. Liu P. Man C. Liu S. (2014). Lactobacillus plantarum NDC 75017 alleviates the learning and memory ability in aging rats by reducing mitochondrial dysfunction. *Exp. Ther. Med.* 8 1841–1846. 10.3892/etm.2014.2000 25371742 PMC4218708

[B189] Phillipson O. T. (2017). Alpha-synuclein, epigenetics, mitochondria, metabolism, calcium traffic, & circadian dysfunction in Parkinson’s disease. An integrated strategy for management. *Ageing Res. Rev.* 40 149–167. 10.1016/j.arr.2017.09.006 28986235

[B190] Pierzchlińska A. Kwasniak-Butowska M. Sławek J. Drozdzik M. Białecka M. (2021). Arterial blood pressure variability and other vascular factors contribution to the cognitive decline in parkinson’s disease. *Molecules* 26:1523. 10.3390/molecules26061523 33802165 PMC8001922

[B191] Poewe W. Seppil K. Tanner C. M. Halliday G. M. Brundin P. Volkmann J. (2017). Parkinson disease. *Nat. Rev. Dis. Primers* 3:17013. 10.1038/nrdp.2017.13 28332488

[B192] Qu Y. Hu H.-Y. Ou Y.-N. Shen X.-N. Xu W. Wang Z.-T. (2020). Association of body mass index with risk of cognitive impairment and dementia: A systematic review and meta-analysis of prospective studies. *Neurosci. Biobehav. R* 115 189–198. 10.1016/j.neubiorev.2020.05.012 32479774

[B193] Quntanilla R. A. Tapia-Monsalves C. (2020). The role of mitochondrial impairment in alzheimer’s disease neurodegeneration: the tau connection. *Curr. Neuropharmacol.* 18 1076–1091. 10.2174/1570159X18666200525020259 32448104 PMC7709157

[B194] Ramalingam A. Wang X. Gabello M. Valenzano M. C. Soler A. P. Ko A. (2010). Dietary methionine restriction improves colon tight junction barrier function and alters claudin expression pattern. *Am. J. Physiol. Cell Ph.* 299 C1028–C1035. 10.1152/ajpcell.00482.2009 20739626 PMC6345211

[B195] Rawat K. Singh N. Kumari P. Saha L. (2021). A review on preventive role of ketogenic diet (KD) in CNS disorders from the gut microbiota perspective. *Rev. Neurosci.* 32 143–157. 10.1515/revneuro-2020-0078 33070123

[B196] Rietdijk C. D. Perez-Pardo P. Garssen J. Wezel R. J. A. V. Kraneveld A. D. (2017). Exploring Braak’s hypothesis of Parkinson’s disease. *Front. Neurol.* 8:37. 10.3389/fneur.2017.00037 28243222 PMC5304413

[B197] Rigottier-Gois L. (2013). Dysbiosis in inflammatory bowel diseases: the oxygen hypothesis. *ISME J.* 7 1256–1261. 10.1038/ismej.2013.80 23677008 PMC3695303

[B198] Rigotto G. Basso E. (2019). Mitochondrial dysfunctions: a thread sewing together alzheimer’s disease, diabetes, and obesity. *Oxid. Med. Cell Longev.* 2019:7210892. 10.1155/2019/7210892 31316720 PMC6604285

[B199] Rinninella E. Cintoni M. Raoul P. Ianiro G. Laterza L. Lopetuso L. R. (2020). Gut microbiota during dietary restrictions: new insights in non-communicable diseases. *Microorganisms* 8:1140. 10.3390/microorganisms8081140 32731505 PMC7465033

[B200] Rolig A. S. Mittge E. K. Ganz J. Troll J. V. Melancon E. Wiles T. J. (2017). The enteric nervous system promotes intestinal health by constraining microbiota composition. *PLoS Biol.* 15:e2000689. 10.1371/journal.pbio.2000689 28207737 PMC5331947

[B201] Romano S. Savva G. M. Bedarf J. R. Charles I. G. Hildebrand F. Narbad A. (2021). Meta-analysis of the Parkinson’s disease gut microbiome suggests alterations linked to intestinal inflammation. *NPJ Parkinson’s Dis.* 7:27. 10.1038/s41531-021-00156-z 33692356 PMC7946946

[B202] Rosario-Alomar M. F. Quiñones-Ruiz T. Kurouski D. Sereda V. Ferreira E. B. Jesús-Kim L. D. (2015). Hydrogen sulfide inhibits amyloid formation. *J. Phys. Chem. B* 119 1265–1274. 10.1021/jp508471v 25545790 PMC4315425

[B203] Sajilata M. G. Singhal R. S. Kulkarni P. R. (2006). Resistant starch–a review. *Compr. Rev. Food Sci. F* 5 1–17. 10.1111/j.1541-4337.2006.tb00076.x 33412740

[B204] Sampson T. (2020). The impact of indigenous microbes on Parkinson’s disease. *Neurobiol. Dis.* 135:104426. 10.1016/j.nbd.2019.03.014 30885792

[B205] Sampson T. Challis C. Jain N. Moisevenko A. Landinsky M. S. Shastri G. G. (2020). A gut bacterial amyloid promotes α-synuclein aggregation and motor impairment in mice. *eLife* 9:e53111. 10.7554/eLife.53111 32043464 PMC7012599

[B206] Sampson T. R. Debelius J. W. Thron T. Janssen S. Shastri G. G. Ilhan Z. E. (2016). Gut microbiota regulate motor deficits and neuroinflammation in a model of parkinson’s disease. *Cell* 167 1469–1480. 10.1016/j.cell.2016.11.018 27912057 PMC5718049

[B207] Schillheim B. Jansen I. Baum S. Beesley A. Bolm C. Conrath U. (2018). Sulforaphane modifies histone H3, unpacks chromatin, and primes defense. *Plant Physiol.* 176 2395–2405. 10.1104/pp.17.00124 29288231 PMC5841731

[B208] Sen P. Molinero-Perez A. O’Riordan K. J. McCafferty C. P. O’Halloran K. D. Cryan J. F. (2021). Microbiota and sleep: awakening the gut feeling. *Trends Mol. Med.* 2020:4. 10.1016/j.molmed.2021.07.004 34364787

[B209] Sender R. Fuchs S. Milo R. (2016). Are we really vastly outnumbered? revisiting the ratio of bacterial to host cells in humans. *Cell* 164 337–340. 10.1016/j.cell.2016.01.013 26824647

[B210] Sharma A. Sethi G. Tambuwala M. M. Aljabali A. A. A. Chellappan D. K. Dua K. (2021). Circadian rhythm disruption and Alzheimer’s disease: the dynamics of a vicious cycle. *Curr. Neuropharmacol.* 19 248–264. 10.2174/1570159X18666200429013041 32348224 PMC8033974

[B211] Shelton C. D. Byndloss M. X. Richardson A. R. (2020). Gut epithelial metabolism as a key driver of intestinal dysbiosis associated with noncommunicable diseases. *Infect. Immun.* 88 e939–e919. 10.1128/IAI.00939-19 32122941 PMC7309626

[B212] Shen L. Liu L. Ji H.-F. (2017). Alzheimer’s disease histological and behavioral manifestations in transgenic mice correlate with specific gut microbiome state. *J. Alzheimers Dis.* 56 385–390. 10.3233/JAD-160884 27911317

[B213] Shen T. Yue Y. He T. Huang C. Qu B. Lv W. (2021). The association between the gut microbiota and Parkinson’s disease, a meta-analysis. *Front. Aging Neurosci.* 13:40. 10.3389/fnagi.2021.636545 33643026 PMC7907649

[B214] Shen X. Yang H. Wu Y. Zhang D. Jiang H. (2017). Meta-analysis: association of *Helicobacter* pylori infection with Parkinson’s diseases. *Helicobacter* 22:e12398. 10.1111/hel.12398 28598012

[B215] Shi Y. Holtzman D. M. (2018). Interplay between innate immunity and Alzheimer disease: APOE and TREM2 in the spotlight. *Nat. Rev. Immunol.* 18 759–772. 10.1038/s41577-018-0051-1 30140051 PMC6425488

[B216] Shlevkov E. Schwarz T. L. (2017). *Axonal Mitochondrial Transport Parkinson’s Disease.* San Diego: Academic Press, 113–137.

[B217] Shoaie S. Karlsson F. Mardinoglu A. Nookaew I. Bordel S. Nielsen J. (2013). Understanding the interactions between bacteria in the human gut through metabolic modeling. *Sci. Rep.* 3 1–10. 10.1038/srep02532 23982459 PMC3755282

[B218] Shoaie S. Lee S. Almeida M. Bidkhori G. Pons N. Onate F. (2021). Global and temporal state of the human gut microbiome in health and disease. *Under Rev* 2021:339282. 10.21203/rs.3.rs-339282/v1

[B219] Silva Y. P. Bernardi A. Frozza R. L. (2020). The role of short-chain fatty acids from gut microbiota in gut-brain communication. *Front. Endocrinol.* 11:25. 10.3389/fendo.2020.00025 32082260 PMC7005631

[B220] Singh S. B. Lin H. C. (2015). Hydrogen sulfide in physiology and diseases of the digestive tract. *Microorganisms* 3:866. 10.3390/microorganisms3040866 27682122 PMC5023273

[B221] Sivanesan S. Chang E. Howell M. D. Rajadas J. (2020). Amyloid protein aggregates: new clients for mitochondrial energy production in the brain. *FEBS J.* 287 3386–3395. 10.1111/febs.15225 31981301

[B222] Snoek S. A. Verstege M. I. Boeckxstaens G. E. van den Wijngaard R. M. de Jonge W. J. (2010). The enteric nervous system as a regulator of intestinal epithelial barrier function in health and disease. *Expert Rev. Gastroent.* 4 637–651. 10.1586/EGH.10.51 20932148

[B223] Sokolov A. S. Nekrasov P. V. Shaposhnikov M. V. Moskalev A. A. (2021). Hydrogen sulfide in longevity and pathologies: inconsistency is malodorous. *Ageing Res. Rev.* 67:101262. 10.1016/j.arr.2021.101262 33516916

[B224] Soscia S. J. Kirby J. E. Washicosky K. J. Tucker S. M. Ingelsson M. Hyman B. (2010). The Alzheimer’s disease-associated amyloid beta-protein is an antimicrobial peptide. *PLoS One* 5:e9505. 10.1371/journal.pone.0009505 20209079 PMC2831066

[B225] Soultoukis G. A. Partridge L. (2016). Dietary protein. metabolism, and aging *Annu. Rev. Biochem.* 85 5–34. 10.1146/annurev-biochem-060815-014422 27145842

[B226] Stanley D. Mason L. J. Mackin K. E. Srikhanta Y. N. Lyras D. Prakash M. D. (2016). Translocation and dissemination of commensal bacteria in post-stroke infection. *Nat. Med.* 22 1277–1284. 10.1038/nm.4194 27694934

[B227] Steiner J. A. Quansah E. Brundin P. (2018). The concept of alpha-synuclein as a prion-like protein: ten years after. *Cell Tissue Res.* 373 161–173. 10.1007/s00441-018-2814-1 29480459 PMC6541204

[B228] Stopschinski B. E. Diamond M. I. (2017). The prion model for progression and diversity of neurodegenerative diseases. *Lancet Neurol.* 16 323–332. 10.1016/S1474-4422(17)30037-6 28238712

[B229] Sullivan P. M. (2020). Influence of Western diet and APOE genotype on Alzheimer’s disease risk. *Neurobiol. Dis.* 138:104790. 10.1016/j.nbd.2020.104790 32032732

[B230] Sun Y. Ma C. Sun H. Wang H. Peng W. Zhou Z. (2020b). Metabolism: a novel shared link between diabetes mellitus and Alzheimer’s disease. *J. Diab. Res.* 2020:4981814. 10.1155/2020/4981814 32083135 PMC7011481

[B231] Sun Y. Sommerville N. R. Liu J. Y. H. Ngan M. P. Poon D. Ponomarev E. D. (2020a). Intra-gastrointestinal amyloid-β1–42 oligomers perturb enteric function and induce Alzheimer’s disease pathology. *J. Physiol.* 598 4209–4223. 10.1113/JP279919 32617993 PMC7586845

[B232] Surmeier D. J. Obeso J. A. Halliday G. M. (2017). Parkinson’s disease is not simply a prion disorder. *J. Neurosci.* 37 9799–9807. 10.1523/JNEUROSCI.1787-16.2017 29021297 PMC5637112

[B233] Svensson E. Horváth-Puhó E. Thomsen R. W. Djurhuus J. C. Pedersen L. Borghammer P. (2015). Vagotomy and subsequent risk of Parkinson’s disease. *Ann. Neurol.* 78 522–529. 10.1002/ana.24448 26031848

[B234] Szabo L. Eckert A. Grimm A. (2020). Insights into disease-associated tau impact on mitochondria. *Int. J. Mol. Sci.* 21:21176344. 10.3390/ijms21176344 32882957 PMC7503371

[B235] Tabat M. W. Marques T. M. Markgren M. Löfvendahl L. Brummer R. J. Wall R. (2020). Acute effects of butyrate on induced hyperpermeability and tight junction protein expression in human colonic tissues. *Biomolecules* 10:766. 10.3390/biom10050766 32422994 PMC7277647

[B236] Tan A. H. Lim S.-Y. Mahadeva S. Loke M. F. Tan J. Y. Ang B. H. (2020). *Helicobacter* pylori eradication in parkinson’s disease: a randomized placebo-controlled trial. *Mov. Disord.* 35 2250–2260. 10.1002/mds.28248 32894625

[B237] Thelen M. Brown-Borg H. M. (2020). Does diet have a role in the treatment of alzheimer’s disease. *Front. Aging Neurosci.* 12:473. 10.3389/fnagi.2020.617071 33424583 PMC7785773

[B238] Thevaranjan N. Puchta A. Schulz C. Naidoo A. Szamosi J. C. Verschoor C. P. (2017). Age-associated microbial dysbiosis promotes intestinal permeability, systemic inflammation, and macrophage dysfunction. *Cell Host Microbe.* 21 455–466. 10.1016/j.chom.2017.03.002 28407483 PMC5392495

[B239] Tomlinson J. J. Shutinoski B. Dong L. Meng F. Elleithy D. Lengacher N. A. (2017). Holocranohistochemistry enables the visualization of α-synuclein expression in the murine olfactory system and discovery of its systemic anti-microbial effects. *J. Neural. Transm.* 124 721–738. 10.1007/s00702-017-1726-7 28477284 PMC5446848

[B240] Trumble B. C. Stieglitz J. Blackwell A. D. Allayee H. Beheim B. Finch C. E. (2017). Apolipoprotein E4 is associated with improved cognitive function in Amazonian forager-horticulturalists with a high parasite burden. *FASEB J.* 31 1508–1515. 10.1096/fj.201601084R 28031319 PMC5349792

[B241] Tse J. K. Y. (2017). Gut microbiota, nitric oxide, and microglia as prerequisites for neurodegenerative disorders. *ACS Chem. Neurosci.* 8 1438–1447. 10.1021/acschemneuro.7b00176 28640632

[B242] Tsuang D. Leverenz J. B. Lopez O. L. Hamilton R. L. Bennett D. A. Schneider J. A. (2013). APOE ϵ4 increases risk for dementia in pure synucleinopathies. *JAMA Neurol.* 70 223–228. 10.1001/jamaneurol.2013.600 23407718 PMC3580799

[B243] Tursi S. A. Tükel C. (2018). Curli-containing enteric biofilms inside and out: matrix composition, immune recognition, and disease implications. *Microbiol. Mol. Biol. Rev.* 82:e00028. 00028-18 10.1128/MMBR PMC6298610 30305312

[B244] Uddin M. S. Tewari D. Al Mamun A. Kabir M. T. Niaz K. Wahed M. I. I. (2020). Circadian and sleep dysfunction in Alzheimer’s disease. *Ageing Res. Rev.* 60:101046. 10.1016/j.arr.2020.101046 32171783

[B245] Unger M. M. Spiegel J. Dillmann K.-U. Grundmann D. Philippeit H. Bürmann J. (2016). Short chain fatty acids and gut microbiota differ between patients with Parkinson’s disease and age-matched controls. *Parkinsonism Relat. Dis.* 32 66–72. 10.1016/j.parkreldis.2016.08.019 27591074

[B246] Urosevic N. Martins R. N. (2008). Infection and Alzheimer’s disease: the apoE ε4 connection and lipid metabolism. *J. Alzheimers Dis.* 13 421–435. 10.3233/jad-2008-13407 18487850

[B247] Van Den Berge N. Ferreira N. Gram H. Mikkelsen T. W. Alstrup A. K. O. Casadei N. (2019). Evidence for bidirectional and trans-synaptic parasympathetic and sympathetic propagation of alpha-synuclein in rats. *Acta Neuropathol.* 138 535–550. 10.1007/s00401-019-02040-w 31254094 PMC6778265

[B248] van Exel E. Koopman J. J. E. Bodegom D. V. Meij J. J. Knijff P. D. Ziem J. B. (2017). Effect of APOE ε4 allele on survival and fertility in an adverse environment. *PLoS One* 12:e0179497. 10.1371/journal.pone.0179497 28683096 PMC5500260

[B249] Van Gerven N. Van der Verren S. E. Reiter D. M. Remaut H. (2018). The role of functional amyloids in bacterial virulence. *J. Mol. Biol.* 430 3657–3684. 10.1016/j.jmb.2018.07.010 30009771 PMC6173799

[B250] Vezzani B. Carinci M. Patergnani S. Pasquin M. P. Guarino A. Aziz N. (2020). The dichotomous role of inflammation in the CNS: a mitochondrial point of view. *Biomolecules* 10:1437. 10.3390/biom10101437 33066071 PMC7600410

[B251] Vidakovic L. Singh P. K. Hartmann R. Nadell C. D. Drescher K. (2018). Dynamic biofilm architecture confers individual and collective mechanisms of viral protection. *Nat. Microbiol.* 3 26–31. 10.1038/s41564-017-0050-1 29085075 PMC5739289

[B252] Vieira M. N. N. Lima-Filho R. A. S. De Felice F. G. (2018). Connecting Alzheimer’s disease to diabetes: underlying mechanisms and potential therapeutic targets. *Neuropharmacology* 136 160–171. 10.1016/j.neuropharm.2017.11.014 29129775

[B253] Villumsen M. Aznar S. Pakkenberg B. Jess T. Brudek T. (2019). Inflammatory bowel disease increases the risk of Parkinson’s disease: a Danish nationwide cohort study 1977–2014. *Gut* 68 18–24. 10.1136/gutjnl-2017-315666 29785965

[B254] Vogt N. M. Kerby R. L. Dill-McFarland K. A. Harding S. J. Merluzzi A. P. Johnson S. C. (2017). Gut microbiome alterations in Alzheimer’s disease. *Sci. Rep.* 7:13537. 10.1038/s41598-017-13601-y 29051531 PMC5648830

[B255] von Martels J. Z. H. Sadabad M. S. Bourgonje A. R. Blokzijl T. Dijkstra G. Faber K. N. (2017). The role of gut microbiota in health and disease: in vitro modeling of host-microbe interactions at the aerobe-anaerobe interphase of the human gut. *Anaerobe* 44 3–12. 10.1016/j.anaerobe.2017.01.001 28062270

[B256] Vuong H. E. Yano J. M. Fung T. C. Hsiao E. Y. (2017). The microbiome and host behavior. *Annu. Rev. Neurosci.* 40 21–49. 031347 10.1146/annurev-neuro-072116-28301775 PMC6661159

[B257] Wallace D. C. Fan W. (2010). Energetics, epigenetics, mitochondrial genetics. *Mitochondrion* 10 12–31. 10.1016/j.mito.2009.09.006 19796712 PMC3245717

[B258] Wang A. Luan H. H. Medzhitov R. (2019). An evolutionary perspective on immunometabolism. *Science* 363:eaar3932. 10.1126/science.aar3932 30630899 PMC6892590

[B259] Wang X. L. Zeng J. Yang Y. Xiong Y. Zhang Z.-H. Qiu M. (2015). *Helicobacter* pylori filtrate induces Alzheimer-like tau hyperphosphorylation by activating glycogen synthase kinase-3β. *J. Alzheimers Dis.* 43 153–165. 10.3233/JAD-140198 25079798

[B260] Waziry R. Chibnik L. B. Bos D. Ikram M. K. Hofman A. (2020). Risk of hemorrhagic and ischemic stroke in patients with Alzheimer disease: a synthesis of the literature. *Neurology* 94 265–272. 10.1212/WNL.0000000000008924 31949087 PMC7136067

[B261] Wei M. Brandhorst S. Shelehchi M. Mirzaei H. Cheng C. W. Budniak J. (2017). Fasting-mimicking diet and markers/risk factors for aging, diabetes, cancer, and cardiovascular disease. *Sci. Transl. Med.* 9:eaai8700. 10.1126/scitranslmed.aai8700 28202779 PMC6816332

[B262] Weil R. S. Lashley T. L. Bras J. Schrag A. E. Schott J. M. (2017). Current concepts and controversies in the pathogenesis of Parkinson’s disease dementia and Dementia with Lewy Bodies. *F1000 Res.* 6:1604. 10.12688/f1000research.11725.1 28928962 PMC5580419

[B263] Whiteman M. Li L. Rose P. Tan C.-H. Parkinson D. B. Moore P. K. (2010). The effect of hydrogen sulfide donors on lipopolysaccharide-induced formation of inflammatory mediators in macrophages. *Antioxid. Redox. Sign.* 12 1147–1154. 10.1089/ars.2009.2899 19769459 PMC2875982

[B264] Włodarek D. (2019). Role of ketogenic diets in neurodegenerative diseases (Alzheimer’s disease and Parkinson’s disease). *Nutrients* 11:169. 10.3390/nu11010169 30650523 PMC6356942

[B265] Wong Y. C. Krainc D. (2017). α-synuclein toxicity in neurodegeneration: mechanism and therapeutic strategies. *Nat. Med.* 23 1–13. 10.1038/nm.4269 28170377 PMC8480197

[B266] Wu G. Shi Y. Han L. Feng C. Ge Y. Yu Y. (2020). Dietary methionine restriction ameliorated fat accumulation, systemic inflammation, and increased energy metabolism by altering gut microbiota in middle-aged mice administered different fat diets. *J. Agr. Food Chem.* 68 7745–7756. 10.1021/acs.jafc.0c02965 32597175

[B267] Yang J. Y. Lee Y. S. Kim Y. Lee S. H. Ryu S. Fukuda S. (2017). Gut commensal *Bacteroides* acidifaciens prevents obesity and improves insulin sensitivity in mice. *Mucosal. Immunol.* 10 104–116. 10.1038/mi.2016.42 27118489

[B268] Yardeni T. Tanes C. E. Bittinger K. Mattei L. M. (2019). Host mitochondria influence gut microbiome diversity: a role for ROS. *Sci. Signal* 12:eaaw3159. 10.1126/scisignal.aaw3159 31266851

[B269] Yonezawa H. Osaki T. Hanawa T. Kurata S. Zaman C. Woo T. D. H. (2012). Destructive effects of butyrate on the cell envelope of *Helicobacter* pylori. *J. Med. Microbiol.* 61 582–589. 10.1099/jmm.0.039040-0 22194341

[B270] Zaretsky D. V. Zaretskaia M. V. (2021). Mini-review: Amyloid degradation toxicity hypothesis of Alzheimer’s disease. *Neurosci. Lett.* 2021:135959. 10.1016/j.neulet.2021.135959 34000347

[B271] Zhan X. Stamova B. Sharp F. R. (2018). Lipopolysaccharide associates with amyloid plaques, neurons and oligodendrocytes in Alzheimer’s disease brain: a review. *Front. Aging Neurosci.* 10:42. 10.3389/fnagi.2018.00042 29520228 PMC5827158

[B272] Zhang L. Wang Y. Xiayu X. Shi C. Chen W. Song N. (2017). Altered gut microbiota in a mouse model of alzheimer’s disease. *J. Alzheimers Dis.* 60 1241–1257. 10.3233/JAD-170020 29036812

[B273] Zhao C. Strobino K. Moon Y. P. Cheung Y. K. Sacco R. L. Stern Y. (2020). APOE ϵ4 modifies the relationship between infectious burden and poor cognition. *Neurol. Genet.* 6:e462. 10.1212/NXG.0000000000000462 32754642 PMC7357411

[B274] Zhou F. Wang D. (2017). The associations between the MAPT polymorphisms and Alzheimer’s disease risk: a meta-analysis. *Oncotarget* 8:43506. 10.18632/oncotarget.16490 28415654 PMC5522165

